# T-2 and HT-2 Toxins: Toxicity, Occurrence and Analysis: A Review

**DOI:** 10.3390/toxins15080481

**Published:** 2023-07-29

**Authors:** Julie Meneely, Brett Greer, Oluwatobi Kolawole, Christopher Elliott

**Affiliations:** 1 Institute for Global Food Security, National Measurement Laboratory: Centre of Excellence in Agriculture and Food Integrity, Queen’s University Belfast, 19 Chlorine Gardens, Belfast BT9 5DL, UK; brett.greer@qub.ac.uk (B.G.); oluwatobi.kolawole@qub.ac.uk (O.K.); chris.elliott@qub.ac.uk (C.E.); 2 The International Joint Research Center on Food Security (IJC-FOODSEC), 113 Thailand Science Park, Pahonyothin Road, Khong Luang 12120, Thailand; 3 School of Food Science and Technology, Faculty of Science and Technology, Thammasat University, 99 Mhu 18, Pahonyothin Road, Khong Luang 12120, Thailand

**Keywords:** mycotoxins, T-2 toxin, HT-2 toxin, analysis, screening, confirmatory, biosynthesis, occurrence, toxicity, *Fusarium*

## Abstract

One of the major classes of mycotoxins posing serious hazards to humans and animals and potentially causing severe economic impact to the cereal industry are the trichothecenes, produced by many fungal genera. As such, indicative limits for the sum of T-2 and HT-2 were introduced in the European Union in 2013 and discussions are ongoing as to the establishment of maximum levels. This review provides a concise assessment of the existing understanding concerning the toxicological effects of T-2 and HT-2 in humans and animals, their biosynthetic pathways, occurrence, impact of climate change on their production and an evaluation of the analytical methods applied to their detection. This study highlights that the ecology of *F. sporotrichioides* and *F. langsethiae* as well as the influence of interacting environmental factors on their growth and activation of biosynthetic genes are still not fully understood. Predictive models of *Fusarium* growth and subsequent mycotoxin production would be beneficial in predicting the risk of contamination and thus aid early mitigation. With the likelihood of regulatory maximum limits being introduced, increased surveillance using rapid, on-site tests in addition to confirmatory methods will be required. allowing the industry to be proactive rather than reactive.

## 1. Introduction

One of the major classes of mycotoxins posing serious hazards to humans and animals and causing severe economic impact to the cereal industry are the trichothecenes [1]. Trichothecenes are a large family (over 200 toxins) of structurally related compounds produced by a broad range of species of fungi such as *Fusarium*, *Cephalosporium*, *Myrothecium*, *Trichoderma*, *Stachybotrys*, *Spicellum*, *Trichothecium* and others in maize, oats, wheat, barley, rye, rice, walnut and tomato [1,2,3]. The most important of these fungal genera are the *Fusaria* since they occur in many habitats, are a global problem, and produce the greatest range of trichothecenes [3]. Although trichothecenes are the most chemically diverse of mycotoxins, only a few are important to human and animal health. The most common metabolites identified in agricultural produce are diacetoxyscirpenol, monoacetoxyscirpenol, HT-2 toxin, T-2 toxin, neosolaniol, 3-acetyldeoxynivalenol, 15-acetyldeoxynivalenol, deoxynivalenol, nivalenol and fusarenon-x [4].

**Figure 1:**
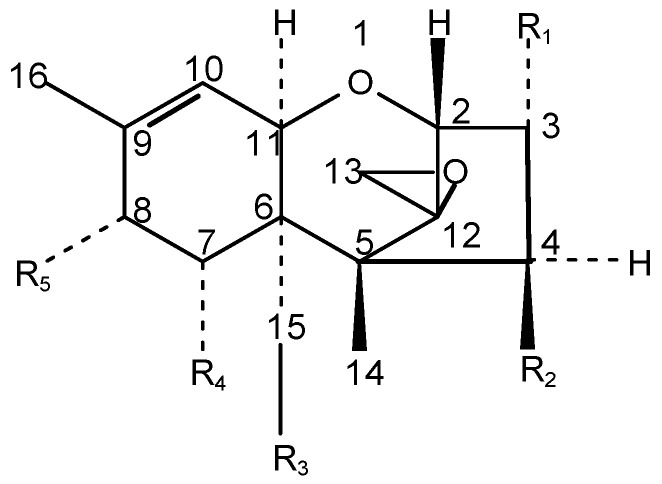
Structure of the trichothecene core.

These toxins are sesquiterpene alcohols or esters containing a 12, 13-epoxide group, essential for toxicity [5,6]. The smaller non-macrocyclic trichothecenes are subdivided into Type A, having a hydrogen or ester type side chain at C-8, and Type B, which has a ketone group at this position. The trichothecene core is outlined in Figure 1. Type-A trichothecenes include T-2 toxin, HT-2 toxin, neosolaniol and diacetoxyscirpenol while nivalenol, deoxynivalenol or vomitoxin and fusarenon-x comprise the Type-B trichothecenes [2]. This review solely focuses on T-2 and HT-2 toxins. Table 1 illustrates the specific side chains of the trichothecenes of interest. 

The trichothecenes are non-volatile low molecular weight compounds (MW 250–500). They are extremely soluble in many solvents, e.g., T-2 toxin and HT-2 toxin are readily soluble in ethyl acetate, acetone and diethyl ether while the group B compounds are polar and very soluble in methanol, acetonitrile and ethanol. Type-A metabolites are remarkably stable to heat and acidic conditions and therefore are not destroyed during normal food processing or digestion; however, they may be destroyed using 3–5% sodium hypochlorite [7,8].

Exposure to these mycotoxins can be through several routes: ingestion of contaminated produce, adsorption through the skin following contact with contaminated grains and inhalation.

The aim of this study was to elucidate the toxicological effects of T-2 toxin and its metabolites in animals and humans; to report the current EU regulations for these toxins; to highlight the specific environmental conditions required for their production, including information on their biosynthetic pathways; to ascertain the impact of climate change on the occurrence and distribution of these toxins; to identify the state-of-the-art methods of analysis for T-2 and HT-2 toxins, in particular, rapid immunological methods and LC-MS/MS; and to expose knowledge or evidence gaps.

## 2. Toxicity of T-2 and HT-2 Toxins

The risks posed by these toxins have been assessed in vitro and in vivo in experimental animals and livestock following the consumption of naturally contaminated feed. There have been a few incidents where humans exposed to these mycotoxins have exhibited toxic infections. These toxins are lipophilic and therefore easily absorbed through skin, gut and pulmonary mucosa [8]. Pharmacokinetics has revealed that regardless of the species tested or the route of administration, T-2 toxin was very rapidly metabolised and excreted in the urine and faeces [8,9]. The International Agency for Research on Cancer (IARC) concluded that as there was limited evidence of carcinogenicity in animals, T-2 toxin is not classified as a human carcinogen, (Group 3) [9].

The typical clinical effects of T-2 toxin through oral, dermal or inhalation exposure include gastric and intestinal lesions, hematopoietic and immunosuppressive effects, anorexia, lethargy, nausea, suppression of reproductive function, hypotension, and shock. Dermal exposure is indicated by skin necrosis and inflammation, oral exposure by lesions in the upper gastrointestinal tract and corneal injury may be observed in those exposed to T-2 aerosols [8].

### 2.1. Toxic Effects in Animals

T-2 and HT-2 toxins can induce acute and chronic effects in animals depending on dosage, the route and duration of exposure, animal sensitivity and the age, sex and health of the animal [10,11]. Clinical symptoms of T-2/HT-2 mycotoxicoses in animals include weight loss, decreased feed conversion and feed refusal, vomiting, diarrhoea, skin problems, haemorrhage, decreased egg production, abortion and death [3,11,12]. Moreover, these toxins are potent inhibitors of protein synthesis and are immunosuppressive [7,13]. Generally, T-2 and HT-2 are more toxic due to their dermatotoxic effect, resulting in necrosis and haemorrhage of the intestinal mucosa [14]. Many studies have been performed on animals to help us understand firstly the toxicity and metabolism of these trichothecenes and secondly their mechanism or mechanisms of action at the cellular level. In 2011, the European Food Safety Authority (EFSA) assessed the lowest observed adverse effect levels (LOAELs) from the available animal studies (Table 2), resulting in a group tolerable daily intake (TDI) of 0.1 µg/kg b.w. for the sum of T-2 and HT-2 toxins being established [15].

In 2017, EFSA published their scientific opinion on the toxicity of these toxins in ruminants. For dairy cows, cereal beef cattle, milking goats and fattening goats, LOAELs of 10 µg T-2 toxin/kg b.w. per day were established, and for lactating sheep a LOAEL of 1 µg T-2 toxin/kg b.w. per day was obtained [16].

Poultry are extremely sensitive to T-2 toxin, and birds suffering from T-2 toxicosis display oral lesions, dermatitis, irritation of the intestines thus leading to loss of appetite or feed refusal [14,17]. Another toxic effect is altered feather structure or “Helicopter disease” [14,18,19]. The consumption of contaminated feed containing 2–6 mg T-2 toxin/kg induced a reduction in feed conversion efficiency, weight gain and feed intake [19,20,21]. Meanwhile, concentrations as low as 0.5–1 mg/kg affected the epithelial cells of the oral mucous membranes, causing oral lesions, indicating the animals are more sensitive to lesions than impaired growth [22,23]. Moreover, as an irritant, T-2 toxin has caused necrosis of the proventricular mucosa and gizzard erosion and is known to cause tibial dyschondroplasia in broilers, a metabolic disease of young poultry that affects the growth of bone and cartilage [14,24].

Additional symptoms observed include decreased egg production, poor shell quality, Raju and regression of ovaries in laying birds [3,14]. A report of T-2 toxicosis on a farm highlighted that egg production was reduced by approximately 22%, the number of cracked eggs increased by 12%, egg breakage was in the region of 18%, incidences of blood spots increased from 0–3% and oral lesions were observed in over 85% of laying hens [14]. Other reported impacts on productivity included poor hatchability and high mortality in goslings and turkey poults, reduced serum total protein and increased concentrations of aspartate aminotransferase (AST) and alanine aminotransferase (ALT) [14,21]. Common immunosuppressive effects of T-2 toxin are leucopoenia, regression of the bursa of Fabricius and increased susceptibility to salmonella infection [3,14,18].

In pigs, haemorrhages on the serosa of the liver, stomach and oesophagus, blood in the intestines and abdominal cavity and a cream-coloured paste on the lining of the oesophagus and the ileum have been reported as a result of exposure to T-2 toxin [24,25]. In addition, various feeding trials have been completed. Animals fed T-2 toxin concentrations of 5–10 mg/kg feed exhibited reduced feed intake and weight gain [26,27]. Reduction in feed intakes have also been seen in 7-week-old piglets fed contaminated feed at a concentration of 0.5 mg T-2/kg feed in addition to decreased plasma leucocytes, confirming the immunosuppressive nature of the toxin [28,29]. In sows, T-2 toxin has been shown to induce infertility and abortion, thus highlighting its endocrine disrupting capabilities [30]. Dermatitis of the nose and at the corners of the mouth are typical symptoms resulting from T-2 exposure [14]. The influence of T-2 toxin on the enteric nervous system, important in the regulatory processes in the gastrointestinal tract and in the adaptive and protective responses to toxins, were summarised in a recent review [31]. The findings suggest that low T-2 exposure might affect digestive motility, secretion, sensory nerve conduction and the regulation of intestinal wall blood flow [31].

While ruminants are reported to be less sensitive to the effects of mycotoxins due to the efficiency of rumen bacteria having the ability to detoxify these toxins [32,33], the available literature suggests that cattle are more sensitive to T-2 toxin compared with other trichothecenes [34]. The main effects described were lesions and haemorrhage in the gastrointestinal tract, enteritis, altered immunity and changes in metabolism [34]. It was postulated that T-2 toxin induced immune suppression in cattle due to a reduction in serum concentrations of IgM, IgG and IgA [35], decreasing neutrophil function and lymphocyte blastogenesis [36]. In addition, the necrosis of lymphoid tissues was shown to be triggered by T-2 toxin [37], and bovine infertility and abortion resulting from consumption of T-2-toxin-contaminated feed has been reported [38]. In calves, the consumption of this toxin at levels of 10–50 mg/kg in feed led to ulceration of the abomasum and sloughing of the papilla in the rumen [39], while dairy cows demonstrated haemorrhagic syndrome after consuming mouldy corn contaminated at 1 mg T-2 toxin/kg feed [40]. Also, as a result of the cytotoxicity of T-2 toxin, severe irritation of the upper respiratory tract and haemorrhagic ruminitis have been reported in cattle following the consumption of contaminated feed [41]. In dairy cattle, the observed effects of T-2 toxin were feed refusal, gastrointestinal lesions, haemorrhagic gastroenteritis, depression, apathy, anorexia hindquarter ataxia and knuckling of the rear feet. Moreover, the oestrus cycle was absent and there was a reduction in milk production [32]. An incidence of poisoning of sheep by the consumption of T-2-contaminated feed described both the acute and chronic effects observed [42]. In the acute phase, sheep were found to be listless, displaying anorexia, ruminal atony and soft faeces and there was a marked reduction in their water consumption. Almost 20% of the sheep died and the animals exhibited rumenitis, ulcerative abomasitis, exocrine pancreatic necrosis, a reduction in white blood cells, inflammation of the heart muscle and oedema of the brain and skin [42]. Chronic pathology revealed the animals presented with weight reduction, reproductive inefficiency, inflammation of the gastrointestinal tract, oral lesions, myocardial fibrosis, immune suppression and altered serum enzymes [42].

In relation to domesticated animals, a study showed that in white rabbits exposed to T-2 toxin over a period of 32 days, serum enzymes concentrations were altered, some liver cell damage was observed and two rabbits died. These results further support the immunotoxicity of this trichothecene. Plasma progesterone levels were affected, thus suggesting reproductive effects [14]. In cats, the administration of T-2 toxin resulted in symptoms similar to those causing alimentary toxic aleukia, a human disease caused by consumption of T-2-toxin-contaminated grains. Clinical observations included vomiting, blood in the faeces, dehydration, weight loss, lethargy, ataxia, shortness of breath and anorexia. Furthermore, bone marrow aplasia, lymphatic tissue alterations, bleeding diathesis, reduced haemostasis and changes in proliferative tissues were demonstrated [14]. In mares dosed orally with T-2 toxin for 32–40 days, oral lesions were observed in three animals, but no reproductive effect was reported [43].

In rats, the immunopathology of low-dose chronic exposure to T-2 toxin was evaluated and the results indicate that both humoral and cell-mediated immune responses were suppressed [44]. In another study in Wistar rats, injury to cardiac tissue was observed on days 28 and 60 following a single injection of T-2 toxin (0.23 mg/kg SC) [45]. To elucidate the mechanisms behind T-2-induced anorexia, changes in the gut satiety hormones peptide YY3-36 (PYY3-36) and glucose-dependent insulinotropic polypeptide (GIP) in plasma were evaluated. Mice were exposed both orally and by intraperitoneal injection of 1 mg/kg bw T-2 and HT-2. The results highlight decreased food intake and elevated PYY336 and GIP concentrations, indicating that these play a role in T-2- and HT-2-induced anorexia [46].

Few studies have been reported regarding the effects of these trichothecenes on fish. Growth impairment, reduced feed intake and dose-dependent depression of haematocrit and haemoglobin concentrations were observed in rainbow trout following a 16-week experiment where they were fed >2.5 mg/kg T-2 toxin. Haemorrhage of the intestine and enlarged gall bladders and spleens were observed in adult trout exposed to 15 mg/kg T-2 toxin [47]. Similarly, low haematocrit values, poor weight gain, reduced feed conversion rations and gastric lesions were demonstrated in catfish fed T-2 toxin [47].

### 2.2. Toxic Effects in Humans

In humans, there have been a few reports of intoxications associated with these trichothecenes. The most notable is alimentary toxic aleukia (ATA), which affected many people (most of which were aged between 10 and 40) in the former U.S.S.R. from 1932 until 1947 and was thought to be due to the ingestion of overwintered grain that contained T-2 toxin and DAS [48,49]. The mortality rate was 60%. Initial exposure resulted in gastroenteritis, gastritis, vomiting, diarrhoea, abdominal and oesophageal pain [2,8,48]. In addition, excessive salivation, headache, dizziness, weakness, fatigue, tachycardia, fever and sweating were presented [8]. A longer exposure of 3–4 weeks caused vertigo, an unpleasant taste in the mouth, leukopenia, granulopenia and progressive lymphocytosis, and if further exposure occurred, the terminal phase developed. This stage was characterised by haemorrhagic diathesis of the nasal, oral, gastric and intestinal mucosa, angina, petechial rash and gangrenous laryngitis leading to aphonia, and death by asphyxia [8,48]. The final recovery stage lasted several weeks to 2 months; however, it was associated with secondary infections such as pneumonia [8,49]. Where the disease outbreak occurred, 5–40% of grain samples showed the presence of *Fusarium sporotrichioides* and *Fusarium poae*, whereas in regions where no disease was present, only 2–8% of grain samples proved positive for these fungi [48]. Subsequently, it has been demonstrated that T-2 toxin was the probable cause [8]. Since the ATA outbreak in the former U.S.S.R., no further human mortalities have been reported due to the consumption of trichothecene-contaminated cereals.

T-2 toxin has been associated with Kashin–Beck disease (KBD), a chronic joint disorder typically found in rural regions of eastern Siberia, northern Korea and in central China. Common symptoms include pain, stiffness and enlargement of the joints accompanied by restriction of movement. Although the aetiology has not been defined, high concentrations of the trichothecene T-2 toxin were reported in the food in areas where the disease was widespread and similar pathological cartilage changes in chicks compared with KBD patients have been observed in experimental studies. Epidemiological studies are required to prove the link between this trichothecene and KBD [50]. Recently, a study performed by Ning et al. [51] identified HT-2 toxin and T-2 tetrol in the urine of adults suffering from KBD when compared with adult controls. Moreover, concentration differences of the metabolite acetyl T-2 toxin were detected in children suspected of having KBD and healthy controls, while different levels of 4-Propanoyl-HT-2 toxin were detected between children with KBD and those suspected of having the disease [51]. Although this metabolomic study has improved the understanding of the aetiology of this disease, further research is needed.

There have also been implications that T-2 toxin has been used in biological/chemical warfare in Afghanistan, Kampuchea and Laos from 1975 to 1984 [52]. Although supported by intelligence reports, epidemiological data and trichothecene analysis of the claims of the “yellow rain” attacks have been discounted in the scientific literature [8]. According to refugee accounts, following exposure to the yellow rain, they experienced severe burning of the skin and began vomiting almost immediately. Additional symptoms included eye pain, blurred vision, headache, dizziness, rapid heartbeat and low blood pressure, chest pain, poor coordination, severe coughing fits, breathing distress, and diarrhoea. Areas of exposed skin broke out in blisters. The mortality rate was between 10% to 20% of those exposed, with death occurring within a few days to a few weeks [52]. Animal deaths including chickens, dogs, pigs, cattle and water buffalo were also reported in addition to contamination and death of crops. Mass spectrometric analysis of leaf and stem fragments marked with yellow spots that were supposedly collected from a battlefield in Cambodia within 24 h after a yellow rain attack was positive for three trichothecenes, as was a sample of yellow powder scraped off foliage in Laos. In total, 6 positive environmental samples and 20 positive human biomonitoring samples led US intelligence to conclude that trichothecenes were being used as biological/chemical agents [8,52]. However, criticism of the method of analysis, control samples used and the absence of any such weapon being found has prevented the unequivocal proof that trichothecenes have been used in biological warfare [8].

## 3. EU/UK Regulations for T-2 and HT-2 Toxins

In 2013, the European Union published their recommendation regarding the presence of T-2 and HT-2 toxins in cereals and cereal products [53]. In infected cereal grains, generally, T-2 toxin will co-occur with HT-2 toxin, and in vivo, T-2 toxin is rapidly hydrolysed to HT-2 toxin [32,53]. For this reason, when performing any risk assessment, the sum of T-2 and HT-2 toxins should be considered.

The Scientific Panel on Contaminants in the Food Chain (CONTAM panel) of the European Food Safety Authority (EFSA) established a group tolerable daily intake (TDI) of 0.1 µg/kg bw for the sum of T-2 and HT-2 [15]. At that time, estimates of human chronic exposure fell below the TDI, therefore the toxins were not deemed an immediate health risk. With respect to animal health, again the risks were considered low for most animals with the exception of cats. Limited data prevented the establishment of a No Observed Adverse Effect Level (NOAEL) or LOAEL; therefore, the recommendation does not include cat food. It was also concluded that there was no evidence of accumulation of these toxins in the tissues of animals fed contaminated feed; thus, human exposure via this route poses no public health concerns [53]. It was also recommended that more data on the occurrence of T-2 and HT-2 toxins in cereals and cereal products be collected in addition to more information on the effects of food processing on the toxins, what factors contribute to high levels of contamination and finally what mitigation strategies could be employed.

Consequently, the European Commission has set indicative limits for these toxins in cereals intended for animal and human consumption. The levels outlined by the EU Commission (2013/165/EU) [53] refer to the sum of T2 and HT2 and are outlined in Table 2. Contamination at or above these levels require further testing to establish an isolated incident. Repetitive findings require identification of the factors contributing to the high levels of contamination [53].

A major complication and topic of huge concern to the food industry is that the EU is currently in discussions with EU member states to set maximum levels for the sum of T-2 and HT-2 in cereals and cereal products. This has been prompted by a re-evaluation of the group TDI in 2017, when it was changed from 0.1 µg/kg bw to 0.02 µg/kg bw for the sum of T-2 and HT-2 [54]. The proposed limits are detailed in Table 3 [55,56].

## 4. T-2 and HT-2 Production

Fungal plant pathogens in the *Fusarium* genus are generally known to be responsible for a number of economically important diseases, including *Fusarium* head blight (FHB) of wheat and barley and ear rot of maize, resulting in huge reduction in the quality and yield of crops [57,58,59]. *F. graminearum* (*Gibberella zeae*), a hemi-biotrophic pathogenic species, is the major *Fusarium* species associated with FHB diseases in many countries. Other *Fusarium* species that have been implicated in the development of FHB disease in small grains include *F. poae*, *F. nivale*, *F. culmorum* and *F. avenaceum* [60]. In addition to the decreases in quality and yield of cereal grains, many *Fusarium* species also have the capacity to biosynthesize toxic metabolites (also known as mycotoxins) under favourable conditions, resulting in the contamination of crops with mycotoxins. T-2 and HT-2 toxins are among the notable mycotoxins produced by toxigenic *Fusarium* species in terms of toxicity and frequent occurrence in food and feed [61,62].

**Figure 2:**
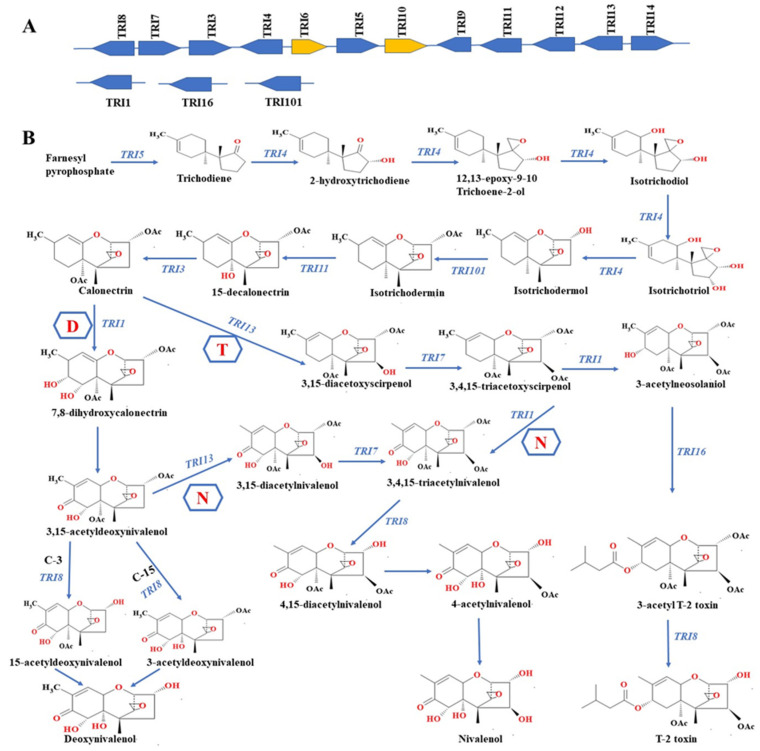
(**A**) Biosynthetic genes that have been reported to be involved in the production of trichothecene by toxigenic *Fusarium* species. The arrowhead shows gene transcription direction, while the arrows in gold represent genes that control the cluster. (**B**) Trichothecene biosynthesis pathways. T represents T-2 toxin biosynthetic pathway; N represents nivalenol biosynthetic pathway; and D represents deoxynivalenol biosynthetic pathway. Reproduced with permission from Kolawole et al. [75] “A review of mycotoxin biosynthetic pathways: associated genes and their expressions under the influence of climatic factors”; published by Fungal Biology Reviews, 2021.

Hundreds of trichothecenes have been reported to date, and they are classified into four classes or types (A, B, C and D) based on their molecular structure [63]. However, type A, including HT-2 and T-2 toxins, and B (3-acetyldeoxynivalenol, nivalenol (NIV), and deoxynivalenol (DON)) are the most prevalent in agricultural commodities worldwide [1,64]. To date, around fifteen genes have been demonstrated to participate in the biosynthetic pathways of T-2 and HT-2 (Figure 2A). Mevalonate and methylerythritol 4-phosphate-independent pathways generally lead to the biosynthesis of a number of compounds, including Farnesyl pyrophosphate (FFP). FFP has been reported to be the main substrate for the biosynthesis of trichothecene compounds [65,66]. A gene (*TRI5*) encoding trichodiene synthase enzyme cyclises FFP to yield trichodiene [65,67]. Subsequently, other genes, including the *TRI101*, *TRI4*, *TRI11* and *TRI3* genes, sequentially catalyse the transformation of trichodiene compound to calonectrin [65,67] (Figure 2B).

Mycotoxin-producing *Fusarium* species generally utilise the same biochemical pathways to produce calonectrin (Figure 2B). Nevertheless, different levels and types of trichothecene compounds can be produced depending on the *Fusarium* strain, crop substrate and climatic zone [68]. For DON-producing *Fusarium* strains, 7,8-dihydrocalonectrin is produced directly from calonectrin catalysis before the production of DON [69]. Meanwhile, in *F. graminearum* strains that biosynthesize NIV, *TRI7* and *TRI13* are the major genes associated with the production of NIV; these fungal strains do not produce DON [70,71]. Additionally, as depicted in (Figure 2B), NIV and its associated derivatives can be produced using 3,15-acetyl DON as a precursor [71,72].

With regard to the biosynthesis of HT-2/T-2 by toxigenic *F. graminearum* strains, the alternative version of the *TRI1* gene has been demonstrated to be responsible for the different structural variations between Type A and Type-B trichothecenes [72,73]. The *TRI1* gene catalyses 3,4,15-triacetoscirpenol, leading to the production of 3-acetylneosolaniol. Subsequently, 3-acetyl-T-2 toxin is produced from 3-acetylneosolaniol through the catalytic activity of C-8 acyltransferase enzyme (encoded by *TRI16* gene). Finally, *TRI8* gene deacetylates 3-acetyl-T-2 toxin, resulting in the production of T-2 (Figure 2B) [74]. HT-2 is formed following the hydrolysis of T-2′s acetyloxy group at position 4S.

## 5. Worldwide Occurrence of T-2 and HT-2 in Oats and Barley

The data available on the global prevalence and concentrations of T-2 and HT-2 toxins in food and animal feed indicate considerable variations from region to region [76,77]. Factors such as temperature, agricultural practices and moisture markedly affect fungal growth and the colonisation of cereals, leading to varying concentrations of these toxic metabolites (especially T-2 and HT-2) across different climatic zones [78,79,80]. For instance, in North America and other Asian countries, incidences of FHB are very high in small grain cereals (barley, maize and wheat), resulting in the high accumulation of key *Fusarium* mycotoxins including deoxynivalenol, zearalenone, T-2 and HT-2 and fumonisins [81]. However, in Northern and Western European countries, oats are less susceptible to FHB [82,83]. Nonetheless, previous mycotoxin surveys have shown that oat grains from this region are frequently contaminated with high levels of HT-2/T-2 toxins compared to tropical regions and other cereals [76,78,79,84] (Table 4).

A survey of milling oats in Scotland in 2019 revealed high prevalence values of T-2/HT-2 toxins in organic and conventional oats of 100% and 83%, respectively [85]. The levels of the sum of T-2 and HT-2 in conventional oats were found to range between 8 µg/kg and 3474 µg/kg, with 19% exceeding the EU indicative limits. In contrast, the highest concentration of T-2 + HT-2 in organic oats was determined as 571 µg/kg [85].

In Russia and West Siberia, oat sample analysis conducted in 2018 and 2019 revealed that HT-2 (75%) and T-2 (55%) were prevalent at the maximum concentrations of 131 µg/kg and 108 µg/kg, respectively, in Russia. Similarly, in West Siberia, the prevalence values of T-2 and HT-2 were 60% and 50%, respectively, and maximum concentrations of 14 µg/kg (T-2) and 9 µg/kg (HT-2) were found [86]. In contrast, barley samples analysed at the same time in these regions revealed relatively high concentrations of T-2 and HT-2 toxins [86]. In barley samples from Russia, T-2 and HT-2 levels ranged from 12–2652 µg/kg and 34–481 µg/kg, respectively, while the levels of T-2 and HT-2 detected in barley samples from West Siberia were between 15–29 µg/kg, and 32–146 µg/kg, respectively [86].

More than 450 oat samples collected between 2002 and 2005 from various cereal fields across the UK were found to contain high prevalence of T-2 (84%) and HT-2 toxins (92%) at an average and maximum concentrations of 570 µg/kg and 9990 µg/kg, respectively, for the sum of T-2 and HT-2 [87]. In addition, unprocessed oat samples in the UK collected over a three-year period (2006 to 2008) were found to be contaminated with T-2 and HT-2 at a mean concentration of 450 µg/kg for the sum of T-2 and HT-2 toxins [84]. Irish oat samples analysed for multiple mycotoxins also revealed a high occurrence of T-2 and HT-2 toxins, with an average concentration of 770 µg/kg [78].

A three-year monitoring (2013–2015) of *Fusarium* mycotoxins in Swiss oat grains harvested at different times showed annual variations (65–76%) in T-2/HT-2 toxin prevalence, with mean and maximum concentrations of 1091 µg/kg and 3789 µg/kg, respectively [88]. Significant levels of T-2 and HT-2 toxins were also found in oats from Norway [89], Finland [90] and Sweden [91]. Conversely, the mean of the sum of T-2/HT-2 toxins concentrations (87.9 µg/kg) in unprocessed Canadian oats collected between 2017 and 2018 were significantly lower compared to the levels of T-2/HT-2 toxins detected in European oats [76,92]. A summary of the prevalence and concentrations of sum of T-2 and HT-2 in oats and barley collected from various countries or regions is presented in Table 4.

**Table 4:** Worldwide occurrence of T-2 and HT-2 toxins in oats and barley.

Region	Year	Commodity	Number of Samples	Positive Samples (%)	Average (µg/kg)	Range (µg/kg)	Reference
Egypt	2021	Oats	10	70	35.4	14.3–74.4	[93]
Canada	2016–2018	Oats	168	81	39.0	10–1155.2	[81]
Croatia	2017–2018	Oats	30	70	87.9	9.5–21.8	[94]
Croatia	2017	Oats	6	75	69.0	23–142	[95]
Europe	2013–2019	Oats	281	98	103.1	5.1–1000	[56]
Lithuania	2010-2018	Oats	72	-	182	-	[96]
Finland	2005–2006	Oats	804	100	348	25–17,451	[90]
Norway	2004–2009	Oats	289	76	105.2	10.2–658.1	[89]
Sweden	2004–2018	Oats	164	-	66.0	-	[77]
UK	2006–2008	Oats	303	85	450	10–8399	[84]
Switzerland	2013–2015	Oats	325	76	225.5	10.1–3789	[88]
Ireland	2015–2016	Oats	208	51	256	53–3405	[78]
Ireland	2020	Oats	202	62	138	5–3064	[79]
Hungary	2014–2015	Oats	29	10	56.0	50.1–69.2	[97]
Scotland	2019	Oats	33	91	574.4	Nd—3474	[85]
Lithuania	2015–2018	Oats	62	100	-	15.7–594.6	[98]
Czechia	2022	Oats	52	92	6 (T-2) 27 (HT-2)	<0.2–31 (T-2) <0.2–128 (HT-2)	[99]
Croatia	2017–2018	Barley	66	41	22.6	12.2–52.1	[94]
Croatia	2017	Barley	7	14.3	3.0	-	[95]
Czechia	2018	Barley	152	-	107.7	48–251.3	[100]
Sweden	2004–2018	Barley	177	-	21.0	-	[77]
Czechia	2012–2017	Barley	117	20	-	11.8–199.0	[101]
Italy	2011–2014	Barley	691	32	127.8	26.0–787.0	[102]
Hungary	2014–2015	Barley	29	14	58.0	52.0–79.0	[97]

-: not stated.

## 6. Impact of Climate Change on T-2 and HT-2 Production

The growth, development and biosynthesis of toxic metabolites by mycotoxin-producing *Fusarium* species have been demonstrated to strongly rely on many agronomic and climatic factors [103,104]. In terms of climatic factors, carbon dioxide, temperature and moisture dictate the type and distribution of fungal species and the levels of mycotoxins produced by toxigenic fungal strains [75,105].

Both T-2 and HT-2 toxins are produced by specific *Fusarium* species including *F. acuminatum*, *F. sporotrichioides*, *F. poae*, and *F. langsethiae* [82,106]. In Europe and other temperate regions, *F. sporotrichioides* and *F. langsethiae* have been reported as the major producers of T-2 and HT-2 toxins [106,107,108,109,110,111]. Several in vitro and in vivo studies on climatic factors influencing growth and T-2/HT-2 production by *F. sporotrichioides* and *F. langsethiae* have shown that both species grow within the temperature range of −2 °C to 35 °C and water activity above 0.98_aw_ [82,112]. Furthermore, the optimal conditions for toxin biosynthesis were recorded at 20–30 °C and 0.98–0.99_aw_ [103]. A decrease in fungal growth and toxin production was observed when water stress increased [103]. Previous field experiments also showed that warm and wet weather conditions before anthesis (May to August) favour the infection of oat heads and accumulation of HT-2 +and T-2 toxins by *F. langsethiae* [84,104,113]. Studies have also shown that elevated CO2 levels affect the growth rate of *F. langsethiae*, *F. poae* and *F. sporotrichioides* and the subsequent formation of the mycotoxins T-2 and HT-2 [110,114]. In addition to weather conditions, agronomic practices (organic or conventional), previous crops, tillage and oat variety can also significantly influence HT-2 and T-2 toxin accumulation in oat grains [79,87,108,115].

A considerable body of knowledge regarding the climatic conditions influencing the growth and production of T-2 and HT-2 production has been established. However, there is currently no information on whether infection and toxin production by these *Fusarium* species occur before harvest or during storage. Furthermore, the ecology of *F. sporotrichioides* and *F. langsethiae* as well as the influence of interacting environmental factors on their growth and activation of biosynthetic genes are still not fully understood. By identifying the impact of key factors and their interactions on mycotoxin accumulation and *Fusarium* species growth, a prediction model can be developed to predict the risk of contamination pre- and post-harvest and for earlier interventions. Moreover, as the EU is currently considering setting new legislative limits for both toxins in cereals, there is a need for continuous monitoring, particularly in oats and barley.

## 7. Analytical Methods

Prior to the analysis of a sample, a representative, homogenous food or feed sample must be provided as the toxigenic fungus, and the associated mycotoxins produced are not always uniformly distributed throughout a lot. A 2006 study by Whitaker et al. [116] indicated that the true concentration of a bulk sample lot could not be determined with 100% accuracy or certainty and that sampling must follow a strict protocol. In particular, if a small sample size is selected, the variation in results will increase; therefore, larger sample and sub-sample quantities are preferred to ensure an even distribution of particle size, usually carried out by blending and/or grinding. Despite the sampling issues, detailed sampling protocols specific for mycotoxins in foodstuffs, including *Fusarium*-produced mycotoxins such as T-2 and HT-2 toxins, have been published in the EU [117] and by the Grain Inspection, Packers and Stockyards Administration (GIPSA) of the U.S. Department of Agriculture (www.gipsa.usda.gov) (https://www.ams.usda.gov/sites/default/files/media/MycotoxinHB.pdf accessed 28 July 2023)).

In addition, EU regulation (2013/165/EU) [53], includes guidelines on the performance criteria required for the testing of T-2 and HT-2 toxins, specifying that where there is a lack of fully validated methodology, a ‘‘fitness-for-purpose’’ approach may instead be used. This is in part due to errors in the methodology that can be introduced during analysis, such as those caused by the homogeneity of the sample, stability and recovery of the analytes to be measured, instrument bias, measurement conditions, reagent purity and the skill and experience of the operator.

The analytical protocols are classified and characterised by several facets, such as being fully qualitative or quantitative or semi-quantitative. The type of analysis performed dictates the simplicity of the test, speed of analysis and level of technical skill required to perform the assay, with screening assays such as enzyme-linked immunosorbent assays, (ELISAs), and lateral flow devices, (LFDs), being more simplistic to use and report on, as well as usually being more rapid as they can be applied in the field where an answer may be required as soon as reasonably possible. Meanwhile, fully quantitative methodologies are usually performed on more technological platforms such as liquid chromatography coupled to mass spectrometry (LC-MS) which serves to increase both the speed and analytical complexity, but with a more accurate end result. Furthermore, the use of fully quantitative methods is more expensive than screening methods, leading to greater cost [118].

### 7.1. Extraction Methodologies

To extract toxins from the matrix to be analysed, several techniques can be employed, which include accelerated solvent extraction (ASE), ultrasonic extraction, liquid–liquid or solid–liquid extraction, depending on the matrix to be analysed and the type of analysis to be performed. This usually requires an admixture of organic solvent such as methanol or acetonitrile along with water in various ratios, sometimes acidified with formic acid or acetic acid. The addition of water to the sample helps the organic solvent penetrate the solid matrix, whereas addition of acid helps break some bonds between the mycotoxin and matrix, both of which aid the extraction efficiency [119]. However, the choice of extraction solvent is dependent on several factors, such as the number of analytes in the final methodology, range of polarities of the suite of analytes and their stability in the extraction solvent chosen. In many cases, the extraction of *Fusarium* type-A trichothecenes such as HT-2 and T-2 is performed using an admixture of an organic solvent with water, with ratios of acetonitrile: water between (50:50, *v*:*v*) and (80:20, *v*:*v*), with the addition of 0.3–10% formic or acidic acid [120,121,122,123]. Incidentally, the use of acetonitrile can be substituted for methanol in some cases. Aqueous buffers are also utilised for extraction, mainly with rapid test kits such as ELISAs, LFDs and fluorescence polarisation immunoassays (FPIAs). This is dependent on the clean-up step employed in such tests where use of organic solvents may affect the antibody used. In this case, the extract must be diluted with aqueous buffer before being applied to the test kit [124,125].

Various physical techniques have been reported for the mixing and extraction of the analytes from the sample using the desired extraction solvent, with rotation, sonication or shaking commonly employed for as little as 3 min up to a maximum of 90 min [122,125].

### 7.2. Sample Clean-Up

As well as extracting the analytes of choice from the matrix, one of the main issues is the co-extraction of matrix compounds and the associated matrix effects, which can lead to issues with the analysis. These include, but are not limited to: signal suppression or enhancement (SSE), poor chromatography, false positives due to the presence of isobaric compounds and poor or incomplete recovery of the target analytes [126]. Therefore, after the initial extraction, a sample clean-up step is usually employed in order to remove as much matrix as possible before analysis, which also serves to decrease the limit of detection (LOD) and limit of quantification (LOQ) should this be a requirement. This clean-up step is usually a requirement for confirmatory analytical methods, but not for most screening assays such as ELISAs, LFDs and biosensor assays due to the specificity of the antibody used and sample dilution. However, if the LOD/LOQ is not at a suitable level, some form of clean-up step may be required to aid this [118].

In recent years, many different techniques have been employed to remove or reduce matrix effects, such as solid-phase extraction (SPE), LLE, QuEChERS (quick, easy, cheap, effective, rugged and safe), solid-phase micro-extraction (SPME), immunoaffinity column (IAC) and dispersive liquid–liquid micro-extraction (DLLME), to name a few [119]. Although these methodologies provide good sensitivity due to the removal of unwanted matrix components, their use limits the number of analytes that can be incorporated due to the selectivity and/or specificity of the techniques employed, i.e., the stationary phase or sorbent used in SPE or SPME or the antibody used in IACs. However, in the analysis of HT-2 and T-2, this is not typically an issue, as only two structurally and physio-chemically related compounds are to be analysed, with most IACs being selective for both, and with the same being true for any SPE column chosen. One thing to note, however, is that most LC-MS methods for the analysis of HT-2 and T-2 toxins are generally not specific for those alone, but are usually incorporated alongside numerous other mycotoxins in the analysis of various matrices [120,122,127,128].

#### 7.2.1. Solid-Phase Extraction

Being one of the most universal sample clean-up techniques, SPE is readily used for the extraction and concentration of mycotoxins from various matrices. It is based on the principle of the partitioning of the analytes between the stationary and liquid phase. The sample extract is applied to a pre-conditioned SPE column, with the analyte(s) retained on the column and then washed to remove impurities, with the target analytes eluted and the resulting eluate evaporated to dryness and reconstituted for analysis [118]. This technique affords enrichment of the analytes by their adsorption to the stationary phase; therefore, it is important to choose an appropriate SPE column to improve the selectivity [119]. There are numerous SPE cartridges with varying chemistries available on the market and used in the analysis of HT-2 and T-2 toxins in foodstuffs, which include: the study of wheat and wheat products using Oasis HLB cartridges, the occurrence of type-A trichothecenes in oats and oat products using MycoSep columns, the analysis of cereals using Strata-XL cartridges, the analysis of cereals including oats using Oasis HLB cartridges and the determination of 12 type-A and -B trichothecenes in cereals and cereal-based food using a Bond Elute Mycotoxin column [129,130,131,132,133]. One thing to note from the aforementioned analyses is that none were specific to HT-2 and T-2 only; instead, they are multi-methods analysing a range of mycotoxins including the *Fusarium*-produced type-A trichothecenes.

Similarly to SPE, micro-extraction techniques including stir-bar sorptive extraction (SBSE), solid-phase micro-extraction (SPME), and micro-extraction by packed sorbent (MEPS) are used for sample clean-up prior to analysis. SPME is more commonly employed alongside GC-MS, whereas MEPS, a miniaturised version of SPE, is fully compatible with LC-MS, with the latter now more routinely employed for the analysis of mycotoxins, and in particular the type-A trichothecenes such as HT-2 and T-2. Furthermore, due to their size, SPME and MEPS are more suited to small volumes of biological fluids for the analysis of mycotoxins and their metabolites rather than in foodstuffs [134].

Dispersive magnetic solid-phase extraction (DMSPE) and LC-MS have been reported for the analysis of thirteen mycotoxins, including T-2 and HT-2 toxins in grass samples [135]. Although results indicate that these method provide sensitive results, the drawback for the routine use of these methods in terms of clean-up is the requirement of synthesising the microcomposite; however, researchers have shown that material can be re-used several times with no effect on extraction efficiency [135].

#### 7.2.2. Immunoaffinity Columns

IAC is based on the premise of attaching an antibody to an inert support that binds specifically to the analyte of interest while allowing interfering components to pass through the column. As with SPE, pre-conditioning, removing unwanted impurities and elution of the target analytes are required. However, in this instance, the extract must be aqueous and contain little or no organic solvent due to having a detrimental effect on the antibody–antigen binding event. Depending on the selectivity of the antibody used, IACs generally afford low LOQs in comparison to other sample clean-up techniques. Several commercial companies have produced IACs for the type-A trichothecenes such as HT-2 and T-2 toxins. Examples in the use of IACs before analysis include: the analysis of compound feed, foodstuffs, oats and oat flours using EASI-EXTRACT T-2 and HT-2 immunoaffinity column alongside LC-MS/MS; the analysis of *Fusarium* toxins including both HT-2 and T-2 toxins in cereals and cereal-derived products using a Myco6in1+ column; the use of 11+Myco MS-PREP^®^ with LC-MS/MS for the analysis of regulated mycotoxins in animal feed; and the use of immunoaffinity columns in tandem prior to multi-mycotoxin analysis in food matrices [136,137,138,139,140,141]. One caveat of the last methodology is that in order to retain all the mycotoxins of choice, it is necessary to use two IACs in tandem, which is not particularly cost-effective. However, if analysing the trichothecenes HT-2 and T-2, the use of one would suffice.

The use of IAC as a sample clean-up technique has vastly improved the analysis of trichothecenes; however, it was stated that a major drawback was the cost of the columns, on top of the fact that they only be used once. It has, however, been demonstrated that many IACs produced in-house may be re-used up to 100 times before observing any significant deterioration, which gives them an edge over the use of SPE cartridges [118].

#### 7.2.3. QuEChERS

QuEChERS is another sample clean-up technique that rapidly expanded from use in the analysis of pesticides to use in food for the analysis of mycotoxins. The method itself combines a liquid extraction and salt partitioning, followed by a clean-up step using dispersive SPE (dSPE). As well as being simplistic, it is relatively cheap in comparison to other techniques such as SPE and IAC and has reduced solvent consumption and therefore solvent waste. The use of the dSPE is similar in one sense to that of SPE in that each has a specific chemistry and can limit the number of analytes included in a method. However, with most methods for the analysis of type-A trichothecenes, in particular HT-2 and T-2, this is not an issue. Studies on the use of QuEChERS for the analysis of the type-A trichothecenes HT-2 and T-2 include the investigation of 11 mycotoxin residues in compound feeds, the simultaneous determination of 11 mycotoxins including aflatoxins, fumonisins and T-2 and HT-2 toxins in cereal-derived products, the analysis of plant-based beverages including those derived from oats, and a method for the simultaneous determination of 20 *Fusarium* toxins in cereals including barley via high-resolution liquid chromatography-orbitrap mass spectrometry [121,123,132,142,143].

Furthermore, some studies have taken the QuEChERS technique and modified it to exclude the addition of the dSPE such as PSA after the salting out process. This simplifies the technique and can result in the inclusion of more analytes in the final method, with the trade-off of an extract that is not as free of matrix impurities. Due to the analysis being directed toward HT-2 and T-2 only, the former is not an issue. Some examples of this modified approach in the analysis of mycotoxins include: the analysis of 4 major *Fusarium* mycotoxins including HT-2 and T-2 toxins in oats, the multi-detection of 22 mycotoxins in various animal feeds, the simultaneous determination of 23 mycotoxins in grains, and the analysis of 14 mycotoxins in feed, with the last methodology including an extra lipid removal step [79,144,145].

#### 7.2.4. Other Sample Clean-Up Techniques

A sample clean-up technique that is now commonly employed for the analysis of mycotoxins in feed and foodstuffs is dilute-and-shoot (DnS). This technique simply involves taking an aliquot of the sample extract and diluting before filtration and analysis, typically via LC-MS. There are numerous examples of this in feed and foodstuffs such as those carried out by Sulyok et al. [146] for the analysis of 39 mycotoxins in wheat, the analysis of 295 bacterial and fungal metabolites including HT-2 and T-2 toxins in four model food matrices by [120] and the expansion of this to over 500 mycotoxins and other secondary metabolites in feed, again containing both the target mycotoxins [120,122,146]. However, one thing to note in the use of this technique is that it is not specific to any class of mycotoxins and is more commonly used for the creation of multi-methods, with the matrix being reduced rather than removed, leading to higher LOQs for most analytes. However, it can be useful for certain labs where screening is important. In this instance, the extract can be analysed for the target toxins, such as HT-2 and T-2, but can also be analysed further by using databases for other possible contaminants or adulterants. One caveat of this is that it is difficult to validate multi-methods to any legislation such as EC directive 2002/657 [147] due to the number of analytes and the resulting complexity, something addressed in the paper by Steiner et al. [148] looking at LC-MS-based multiclass methods for the quantification of food contaminants [148].

Another emerging clean-up technique is the use of immunomagnetic beads based on metal–organic framework materials (MOFs). Using MOFs conjugated to monoclonal antibodies allowed for the purification of several mycotoxins including T-2/HT-2 from various flours, with the study comparing this against IAC purification. The results revealed no difference between both clean-up methodologies [149]. Therefore, there is the potential for this to be extended to oats and barley for the clean-up of T-2/HT-2 from the matrices of choice.

### 7.3. Analysis

There are a wide variety of analytical tests available for the analysis of the *Fusarium*-produced HT-2 and T-2 toxins, with these ranging from sophisticated confirmatory/reference methods that can be fully quantitative, to rapid screening assays that are semi-quantitative or qualitative, examples of which are outlined in Table 5.

The confirmatory methods generally used chromatographic separation, with gas chromatography coupled to flame ionisation detection (GC-FID) or mass spectrometry (GC-MS) being the method of choice for the major type-A trichothecenes [150,163]. However, due to the low volatility of these mycotoxins, a derivatisation step after the sample clean-up step is required, which adds to the complexity of the methodology and potential for human error. Of late, this has been superseded by liquid chromatography (LC), whether high-performance liquid chromatography (HPLC) or ultra-high-performance liquid chromatography (UHPLC). These are usually coupled to either high-resolution mass spectrometry (HRMS) or low-resolution mass spectrometry (LRMS), more commonly referred to as a triple quadrupole (MS/MS), with spectrofluorometric (FLR) or ultraviolet (UV) detection also employed [123,125,139]. However, similarly to the use of GC-MS, a derivatisation step is required for fluorescence detection, whereas this is not the case when using LC-HRMS or LC-MS/MS. These aforementioned methods rely on considerable laboratory investment in terms of the equipment, as well as skilled personnel to operate them and interpret the data produced [164]. Therefore, along the supply chain, a more simplistic analytical approach is required that is more user-friendly, inexpensive and rapid. However, these methods must be accurate, reproducible and provide the sensitivity required for regulatory compliance.

Although no legal limits for T-2 and HT-2 mycotoxins have been set in Europe to date, there is a recommendation for the levels in various feed and foodstuffs [53]. However, it is a requirement that analyses must detect the presence of both T-2 and HT-2 toxins in food and feed. To that end, and in order to improve the quality of raw materials such as oats, barley and their products in the agri-food industry, rapid tests have increasingly been utilised on-site for sample analysis and to validate food safety management systems. These rapid tests do not require much, if any, scientific expertise, and can be used as complimentary, high-throughput screening before being sent to a laboratory for confirmatory analysis. With climate change and the resulting increase in mycotoxin contamination on the Island of Ireland and globally, and in particular the T-2 and HT-2 contamination of oats and barley in this instance, there is the need for more routine analysis of these commodities to safeguard human and animal health, as well as to reduce the economic impact on growers and farmers.

In this section, the analytical techniques employed for the analysis of T-2/HT-2 toxins in oats, barley and cereal products are reviewed. They include quantitative, semi-quantitative and qualitative (screening) methodologies, including those validated to EC directive 2002/657 [147] and those considered state-of-the-art. Various analytical methods have been employed and include GC-MS, LC-MS/MS, LC-HRMS as well as the rapid tests, which are mainly immunochemical, such as ELISA, LFDs/dipstick assays, surface plasmon resonance (SPR) biosensors and fluorescence polarisation immunoassays (FPIAs) [165]. The use of some novel techniques including spectroscopy and electrochemical biochip assays are also explored.

#### 7.3.1. Rapid Diagnostics

There are many rapid diagnostic tests on the market for T-2/HT-2 toxins, (Table 6), the majority being immunochemical methods, including ELISAs, LFDs, SPR and FPIA as mentioned above.

The speed of these test kits is particularly important in the agri-food industry, as when raw materials are delivered, it is important that a sample can be tested rapidly for compliance and therefore avoid disruption to the supply chain [166]. Another advantage with rapid diagnostic kits is their ease of use and portability, which are important aspects to consider for producers monitoring regulated mycotoxins such as HT2/T2. The use of such kits facilitates on-site testing, negating the requirement for laboratory equipment or need for skilled laboratory staff, which thereby reduces the cost per analysis and in turn encourages farmers and producers to routinely test [167]. Some of these test kits also use an aqueous buffer or water as the extractant, which is always advantageous when not in a laboratory setting so as to minimise solvent waste and damage to the environment. Therefore, those assays listed in Table 6 that use an aqueous extraction buffer or water-based extraction would be the most advantageous, such as the RIDA^®^QUICK T-2/HT-2 RQS ECO lateral flow device or RIDASCREEN^®^ T-2/HT-2 Toxin ELISA, both from R-Biopharm AG, or the Reveal^®^ Q+ MAX for T-2/HT-2 and Reveal^®^ Q+ for T-2/HT-2 lateral flow devices, both from Neogen Corporation.

However, there are some notable disadvantages associated with use of rapid immunochemical test kits. The most significant of these is the specificity of the antibody used and the associated cross-reactivity and/or matrix effects that interfere with the signal. These more often than not lead to over-estimation and therefore increased measurement uncertainty of the measured concentration, or in some instances false positives [118,164]. One of the most important aspects in a rapid test kit is the specificity or cross-reactivity of the antibody utilised. For example, the metabolites of T-2 toxin, T-2 Tetraol or T-2 triol may cross-react with the antibody, leading to an over-estimation of the levels of T-2, as these metabolites are not included in legislation for food. Conversely, another metabolite of T-2 toxin, HT-2 toxin, is included in the legislation, as the regulatory limits refer to the sum of T-2 and HT-2. Therefore, the antibody must show good specificity for this metabolite in order for an accurate result to be reported, otherwise there could be an under-estimation of results [118,164]. Furthermore, if a test kit is required for a different matrix, a full validation must be performed to ensure it is “fit for purpose” and will provide the necessary accuracy, sensitivity and precision as directed.

ELISA methods are considered the gold standard for screening assays in the food sector, with them routinely employed by end users due them being cost-effective, high-throughput and relatively straightforward to use. Improvements are always being made to those already on the market due to changes in validation criteria or to extend an existing kit to a new food commodity, as most ELISA kits are specific to certain matrices. They are an excellent tool for rapid analysis to assess whether a food commodity is compliant and can enter the food chain. However, as detailed above, they can cross-react with other proteins and/or matrix components, leading to over-estimation and false-positives. Therefore, any sample with a concentration higher than the EU recommended level should be sent to a laboratory for confirmatory analysis.

ELISAs are colorimetric assays performed in microtiter plates, with the assay itself being competitive due to the low molecular weight of the target compounds (<1 kDa). Briefly, a specific antibody to the target analyte, either mono- or polyclonal, is used to coat the wells of a 96-well microtiter plate. Sample is then added to the wells, which is then followed by addition of the target analyte (T2/HT2) that is conjugated to an enzyme. Competition between the unlabelled and labelled antigen (T2/HT2) for antibody binding sites occurs during the incubation period. The plate is then washed to remove unbound material and the enzyme-labelled bound antigen is measured by the addition of an enzyme substrate that reacts to produce a colour change. The absorbance is measured at a specific wavelength using a plate reader, with the absorbance being inversely proportional to the amount of toxin present [164].

As detailed above, any antibody used in an immunochemical assay must show specificity for both T-2 and HT-2, as it is the sum of both that must be reported. However, some of the commercial ELISA kits outlined in Table 5 include kits that show poor cross-reactivity for HT-2, such as those produced by Hygiena LLC (3% for HT-2) or the RIDASCREEN^®^ T-2 Toxin ELISA (R-Biopharm AG) (7% for HT-2), which would under-estimate due to the antibodies’ cross-reactivities. Furthermore, the AgraQuant^®^ T-2 Toxin ELISA test (Romer Labs Diagnostic GmbH), MaxSignal^®^ T-2 ELISA Kits (PerkinElmer) and RIDASCREEN^®^ FAST T-2 Toxin ELISA (R-Biopharm AG) do not specify the cross-reactivity profile and therefore may not be suitable for the testing of both toxins. In fact, a 2013 study carried out by Aamot et al. indicated that ELISA kits that could simultaneously and reliably detect both HT-2 and T-2 were required. Their study to estimate the sum of HT-2 and T-2 in oat samples using the RIDASCREEN assay above indicated that it was necessary to re-calculate data from both ELISA kits from the known cross-reactivities of each kit to obtain the actual concentration, with the R^2^ values for the correlation with the LC-MS/MS confirmatory method being 0.61 and 0.83 for the “Fast ELISA” and “Standard ELISA”, respectively [168].

An alternative commercially available chemiluminescence immunoassay technique known as Biochip Array Technology manufactured by Randox Laboratories Ltd. was developed for the simultaneous detection of seven mycotoxins in animal feed. A single laboratory validation showed ccα and ccβ concentrations of 3.70 µg/kg and 4.84 µg/kg, respectively, for T-2 toxin [169]. Recently, in an inter-laboratory study, the test kit was found to have a 97% Z-score pass rate [170]. However, the cross-reactivity of the antibody is 100% for T-2 toxin and only 37% against HT-2 toxin [169]; therefore, the kit is not applicable to the analysis of the sum of T-2 and HT-2 toxin.

Apart from commercially available ELISA kits, some have been developed in-house that analyse both T2 and HT2. These include a T-2/HT-2 ELISA based on a T-2 monoclonal antibody, indicating a 125% cross-reactivity with HT-2. The newly developed ELISA was validated in accordance with the recent guidelines for the validation of semi-quantitative screening methods for mycotoxins included in Commission Regulation (EC) No 519/2014 [171], and was carried out in several matrices including barley and oats. The accuracy of the ELISA was then confirmed through proficiency testing and reference samples [165]. An HT-2 toxin-specific ELISA assay was developed based on an anti-immune complex (IC) scFv antibody fragment, genetically fused with alkaline phosphatase (AP). The primary antibody recognised both HT-2 and T-2, however the anti-IC antibody made the assay specific to HT-2 only, with the assay performance tested in both barley and oats. However, with the ELISA specific for HT2 only, the EU criteria would not be met. However, the authors stated that a similar assay with anti-IC antibodies could also be developed for T-2 that would allow for an accurate multiplex measurement [124]. The group of Zhang et al. [151] then developed what they referred to as a competitive amplified luminescent proximity homogenous assay (AlphaLISA) using a T-2 antibody, which negates the need for washing the plate and results in a low coefficient of variation. Although the authors did not specify the cross-reactivity of the antibody with HT-2, the detection range of both was identical, with half-maximal inhibitory concentration (IC50) values of 2.28 ng/mL and 2.75 ng/mL for T-2 and HT-2, respectively [151].

Further developments with ELISAs have been made using nanomaterials, such as the use of magnetic nanoparticles (NMPs), which are suspended in the reaction media and used as a solid support for the antibody. This facilitates a greater coverage of the reaction media with antibody, leading to an increased probability of antibody–antigen conjugation and therefore less incubation time [164]. As traditional ELISAs use antibody recognition and HRP-catalysed TMB to generate colour and therefore a signal to determine the concentration present, usually requiring a plate reader, efforts have been made to improve the colorimetric signal. This is due to the signal produced not being suitable for naked-eye detection. Therefore, acid–base indicators have been seen as the best signal generators for eye detection, with various enzymes being utilised to change the pH through catalysis of the substrate to produce hydrogen or hydroxide ions. Another recent advancement is the use of plasmonic ELISA using gold nanoparticles as candidates for colorimetric indicators, with these advancements potentially negating the need for specific plate readers and therefore also being used in the field [172]. However, at present, the majority of these developments have been in the analysis of OTA and AFB1 rather than for HT2/T2. One study by McNamee et al. [173] created a multiplex nanoarray-based ELISA technique via the nano-spotting of mycotoxin–protein conjugates into single wells of a microtiter plate analysing 3 mycotoxins, including T-2, with the assay also detecting HT-2, with the antibody showing 74% cross-reactivity with the method validated in wheat [173]. A further example is the design of a highly-sensitive chemiluminescent ELISA (CL-ELISA) for the detection of T-2 and its metabolite HT-2. The study by Li et al. [174] used an anti-T-2 mAb and a SuperSignal chemiluminescence substrate solution to generate the signal, which showed superior sensitivity compared to traditional colorimetric ELISA substrates previously used, with LODs of 8.84 and 5.62 µg/kg for T-2 and HT-2, respectively. The antibody used also indicated no cross-reactivity with other structurally related mycotoxins, further improving its specificity and therefore use for the detection of the sum of both T-2/HT-2. At present, this study was conducted in rice, but there is the potential to further develop it for use in the matrices of choice, provided the method performance limit can be met [174].

LFDs also use the competitive format as described above for ELISAs. The basic premise of the methodology includes the extracted sample being applied to a sample pad, which then traverses along the membrane. Once the extract reaches the conjugate release pad, the dry conjugate containing the labelled antibody is hydrated, and if analyte is present in the sample extract, it binds to the antibody and continues along the strip. If no antigen (toxin) is present, the free antibody binds to the test line. Therefore, the presence of a coloured line is inversely proportional to the amount of toxin present, with the labelled antibody used as a signal reagent. A positive sample will be saturated from toxin present in the sample, and no visible line will be present. Conversely, a negative sample will have a visible line in the “test zone”, with the line visibility dependent on the degree of sample contamination. LFDs will have a cut-off level, which is the point of discrimination between a positive and negative sample, and this level must meet the regulatory requirements for the maximum permissible level [164]. These LFDs can either be qualitative or quantitative, with the latter providing a concentration after the test, in which case some sort of reader must be used, with the sensitivity of LFDs having the ability to match that of an ELISA, and as with an ELISA, the antibody used must have cross-reactivity for both HT-2/T-2, as it is the sum that must be reported.

There are commercial LFDs on the market for the analysis of HT-2/T-2 in the cereals of choice, oats and barley. Of those, there were only a few that meet the requirements: the ROSA T-2 and HT-2 Quantitative Test (Charm Sciences Inc., Lawrence, MA, USA), T-2(T-2 Toxin) Lateral Flow Assay Kit (Elabscience Inc.) and the T2/HT2-V AQUA, (Vicam LP). However, there are other LFDs on the market not validated in both matrices, such as the Reveal^®^ Q+ MAX for T-2/HT-2 (Neogen Corporation) and the RIDA^®^ QUICK T-2/HT-2 RQS ECO (R-Biopharm AG), which have only been validated for oats. Out of all the LFDs that have been validated in both matrices, barley and oats, the ROSA T-2 and HT-2 Quantitative Test (Charm Sciences Inc.) and the T2/HT2-V AQUA (Vicam LP) appear the most attractive due to incorporating a water-based extraction. The EU Commission has stated that the method LOQ should not exceed 10 µg/kg for T-2 and HT-2 individually and that the LOD should be ≤25 µg/kg for the sum of T-2 and HT-2 [53]. In regard to the ROSA T-2 and HT-2 Quantitative Test produced by Charm Sciences Inc., they specify a quantification range of 25–200 µg/kg rather than stating the LOD of the method. Furthermore, LFDs produced by Neogen Corporation, R-Biopharm AG and PerkinElmer Inc. state LODs of 50 µg/kg, and while these meet the standard required for determination of the sum of T-2/HT-2 in unprocessed cereals, they are not suitable for cereal products or cereal-based infant and children food where the limits are 25 µg/kg and 15 µg/kg, respectively. However, unlike ELISA kits, LFDs can be used on-site to test raw materials as they do not require a microplate reader or spectrophotometer because the absence or presence of a visible line indicates a sample that is either non-compliant or compliant, respectively.

As well as commercially available LFDs, there are also those produced in-house for research purposes and to extend their capabilities. One such example is that of Foubert et al. [175], who developed a multiplex lateral flow immunoassay (LFIA) for the determination of four mycotoxins, including both T2/HT2 toxin in barley using differently coloured luminescent quantum dots (QDs) as labels. The T2 antibody utilised showed 110% cross-reactivity with HT-2, with the assay showing good sensitivity, being rapid (15 min) and having a low false-negative rate (<5%). The same group also compared the use of colloidal gold nanoparticles (CG) to quantum dots (QDs) as labels for the LFIA, indicating that the QDs gave better sensitivity in comparison and that the results were easier to interpret [176].

Fluorescence polarisation immunoassays (FPIAs) use fluorescence for the detection of the analytes of choice, with the use of both excitation and emission wavelengths. The principle is based on the fact that when a fluorophore in solution is exposed to plane-polarised light at its excitation wavelength, the subsequent emission is depolarised. The format of this methodology is a competitive assay, whereby toxin (T2/HT2) is covalently linked to a fluorophore to make a fluorescent tracer molecule. The tracer molecule then competes with toxin in the sample extract (if present) for a limited amount of toxin-specific antibody. In the absence of any toxin(s) being present, the antibody binds the tracer, resulting in high polarisation. Conversely, if there is toxin(s) present in the sample extract, less of the tracer molecule will bind the antibody and a greater fraction exists unbound, resulting in lower polarisation.

The results are read using instruments that can determine the amount of fluorescence polarisation and, therefore, amount of toxin(s) present, with the degree of polarisation being inversely proportional to the mycotoxin concentration. This method does not require separation between free and bound tracer and therefore no washing steps are required, negating the incubation step as no colour development is required, therefore reducing assay time and increasing throughput. One caveat of no washing step is that it can lead to matrix effects that can cause interference in analysing the results. In order to reduce or eliminate these interferences, some sort of pre-treatment may be required in order to prevent an overestimation of the toxin level, with this adding to the assay time [164]. There are numerous test kits available based on FPIA; however, the majority to date are not for the determination of T2/HT2 but for other mycotoxins such as DON, aflatoxins and fumonisins. They have, however, been validated in the matrices of choice for these analytes (oats and barley) and therefore there is the potential for them to be tailored to the analytes of choice [177].

An example in the use of FPIA for the mycotoxins of choice is that by Lippolis et al. [153]. In their study, they developed a FPIA using an HT-2 specific antibody with 100% cross-reactivity for T-2 toxin using two differing extraction protocols, one organic and one aqueous, for the analysis of both toxins in wheat. Their FPIA had an LOD of 10 µg/kg for both toxins and a false positive rate of <0.1%, meeting the criteria for acceptability of an analytical method for quantitative determination of T-2 and HT-2 as laid down by the EU [171]. Both methods were validated in accordance with the guidelines for validation of screening methods included in Regulation (EC) No. 519/2014 [171]. Apart from not being applied to the matrices of choice, the main flaw in the design was that the antibody used showed high cross-reactivity (80%) for both T-2 and HT-2 glucosides, although this was part of the study. However, if any of these glucosides were present in a sample, the overall level reported would be elevated and could potentially breach the permitted level, even though the sum of HT-2/T-2 may well be below this [153]. This may be relevant as the glucosides could potentially be converted back to their native form upon ingestion and contribute to the overall toxicity; however, at present, the regulations are for the sum of HT-2/T-2 only.

Biosensors are composed of two elements: the molecule that reacts with the analyte of choice and the transducing element that converts the physical change into a measurable signal. The recognition element is usually an antibody–antigen, enzyme–substrate or receptor-biospecific molecule, with the transducer usually optical or electrochemical [178]. In the former, an antibody specific to the toxin(s) is mixed with the sample extract before being applied to the chip (sensor surface) so that any free mycotoxin(s) in the sample will bind to the antibody, which results in no free antibody binding to the probe on the sensor surface. Conversely, if a sample is negative, the antibody is free and will therefore bind to the probe on the sensor surface. Any binding changes the resonance frequency of the surface plasmons, with this resulting in a change in intensity of the reflected light, detected by the biosensor device [164]. SPR-based biosensors are considered reliable, sensitive and have the added advantage of reusability with regeneration of the biosensor chip surface and are also quantitative, as they can be run against a calibration curve to determine the concentration.

Surface plasmon resonance (SPR) has been used in the analysis of mycotoxins, with examples of its use for the analysis of the mycotoxins of interest, including the determination of the sum of HT-2/T-2 in various cereals and cereal-based baby foods [179,180], with the latter study by Meneely et al. [179] being multiplex as it included another trichothecene, DON. Importantly, there was no cross-reactivity of DON with the HT-2 antibody used and vice versa. In 2011, an ultrasensitive method for the detection of T-2 was developed through the combination of a molecularly imprinted polymer (MIP) with SPR, with MIPS displaying high selectivity and specificity to a particular analyte. The LOD of the assay was 0.05 pg/mL, making this an extremely sensitive method; however, there were no details on the cross-reactivity of this T-2-MIP with HT-2 [181].

A 2014 review by Meneely et al. [182] indicated the need to develop and manufacture portable and multiplex SPR instruments, although in the case of HT-2/T-2, multiplexing is not an issue. Further developments since then have included the study carried out by Joshi et al. [154], who developed a multiplex competitive inhibition immunoassay using a portable nanostructured imaging surface plasmon resonance (iSPR) instrument for the detection of 6 mycotoxins in barley including T-2, with the T-2 antibody showing a cross-reactivity of 76% with HT-2. The LOD for T-2 was calculated as 0.6 µg/kg, with an in-house validation indicating that T-2 could be detected at the European Union regulatory limits. This study therefore highlighted the potential of this prototype for rapid on-site screening for mycotoxins. One thing to note from this study was that analysis of naturally contaminated barley samples using this assay gave a T-2 level of 46% less than the known value, most likely due to incomplete extraction of the analyte from the matrix [154]. A further study by Hossain et al. [183] developed an iSPR assay for the detection of T-2 and its glucoside, albeit in wheat rather than oats or barley. The antibody used showed <1% cross-reactivity with HT-2 and therefore would not be suitable for on-site screening due to not being able to report on the sum of T-2/HT-2 [183].

SPR biosensors from Biacore AB have been demonstrated as applicable for mycotoxin testing, with SPR biosensors widely used in academic research. However, there are issues in regard to their commercialisation for mycotoxin analysis due to the data analysis requiring technical expertise, and the miniaturisation of such instrumentation needs optimisation in order to maintain high sensitivity, with both of these limiting the ability to be used on-site. Furthermore, although the developments noted above had led to improvements in portability and sensitivity, the reagents used in order to achieve this add to the overall cost of any potential commercial product.

The use of spectroscopy for the analysis of mycotoxins has been explored, with the majority of these being non-invasive/non-destructive and therefore maintaining the integrity of the sample. These techniques include near-infrared reflectance spectroscopy (NIR), Fourier transform infrared spectroscopy (FTIR) and surface-enhanced Raman spectroscopy (SERS). In essence, whichever technique is chosen involves light of specific wavelengths being shone on the surface of the food matrix, with this generating spectral data. In order to interpret the spectra, models must be built in order to assess the data generated due to the interpretation of spectral data being difficult as well as many of the spectra overlapping. One emerging technique in this area is the use of surface-enhanced Raman spectroscopy (SERS), with there being an increasing interest in the use of SERS for mycotoxins analysis due to the availability of appropriate nanostructures for substrates, which is required for this technique. When using SERS, the type of substrate used is crucial for analyte detection due to the substrate determining the signal enhancement, sensitivity, selectivity and reproducibility, with the substrate loosely divided into two groups: colloidal substrates (silver or gold) and solid-surface-based substrates. The latter reduces the high variance seen with colloidal substrates, generating a highly reproducible and long-term-stable substrate [184]. In order to use SERS, and unlike other spectroscopy techniques, the sample must be extracted; LLE or SLE are often used depending on the matrix. This also extracts unwanted matrix components that may interfere with the spectral data and therefore need to be removed or reduced, with this achieved through the use of techniques such as SPE, IAC and QuEChERS. To date, although there are some studies using spectroscopy for the analysis of mycotoxins, there are very few focused on HT-2/T-2. One example is the multiplex SERS-based lateral flow immunosensor assay developed by Zhang et al. [185] to detect 6 mycotoxins including T-2, with an LOD of 8.6 pg/mL for T-2 toxin. The monoclonal antibody against T-2 also had a cross-reactivity of 119% for HT-2, indicating that it meets the criteria for screening since both toxins can be measured [185].

More recently, Maragos [152] reported the application of portable mass spectrometry (atmospheric pressure chemical ionisation (APCI-MS)) to the screening of T-2 toxin in wheat and maize. T-2 concentrations above 200 µg/kg and HT-2 concentrations above 900 µg/kg were detectable; however, in its current form, this method would not satisfy regulations. That said, it is an exciting development in the monitoring of these toxins and further research may result in increased sensitivity [152].

#### 7.3.2. Confirmatory Analysis

Although the techniques discussed above are useful, especially for on-site screening, any sample that is non-compliant after a screening test must undergo confirmatory analysis. This can be performed using GC or LC (HPLC/UHPLC) as the separation technique, with these usually coupled to a detector with methods such as flame ionisation detection (FID), mass spectrometry (MS), a photodiode array (PDA) using ultraviolet light (UV) or fluorescence detection (FLR). The first two entries in this list have been used extensively in the analysis of mycotoxins, with gas chromatography coupled to mass spectrometry (GC-MS) having been the method of choice for the major type-A trichothecenes such as T-2/HT-2. However, as mentioned previously, a derivatisation step is required due to their low volatility, which adds to the complexity and potential for error in the methodology. Similarly for analysis using LC-FLR, a derivatisation step is required, whereas this is not the case when using LC-UV or LC-MS. HPLC coupled to UV/PDA or FLR has also been frequently used in the analysis of Type-A trichothecenes, with the latter requiring pre- or post-column derivatisation prior to analysis, while use of UV/PDA detection is generally not employed due to the lack of a strong chromophore in both molecules and therefore only being applicable for samples with high concentrations of these compounds, potentially leading to false negatives. At present, there is no standardised or official method for the analysis of T-2/HT-2 in food and/or feed, but it is a recommendation that member states monitor and report on both T-2 and HT-2 in food and feed. Furthermore, the LC-MS method must be validated in-house to evaluate its performance, ensuring it is compliant with the acceptability criteria specified in Commission Regulation 401/2006/EC [117] to ensure it is fit for purpose. Of late, LC-MS has become the gold standard and most extensively used technique for confirmatory analysis, with both triple-quadrupole (QqQ) and high-resolution (HRMS) detectors both used in LC-MS/MS and LC-HRMS, respectively [118]. These systems offer high selectivity, specificity and low detection levels and can be used to analyse multiple analytes in a short period of time.

An example in the use of GC-MS is that of Pereira et al. [150], who simultaneously analysed 12 trichothecenes in cereal-based baby foods, using QuEChERS as the clean-up step. Their method indicated LODs of 6.4 and 6.76 µg/kg for HT-2 and T-2, respectively, with a total run time of just over 21 min [150]. Another study utilising GC was that by Carballo et al. [155], who analysed 27 mycotoxins including HT-2 and T-2 in ready-to-eat foods including some cereal-based ones using GC-MS/MS, with some of the analytes detected using LC-MS/MS. Their method used a QuEChERS-based methodology and indicated LODs of 0.75 and 0.15 µg/kg for T-2 and HT-2, respectively, for the cereal-based products, with a run-time of just over 20 min [155]. HPLC-UV/PDA and HPLC-FLR have also been utilised in the analysis of T-2 and HT-2. Examples of this include the study carried out by Soleimany et al. [186] analysing 12 mycotoxins in cereals including T-2 and HT-2, with LODs of 9.4 and 6.2 µg/kg, respectively. However, the instrument setup was quite complex as it combined HPLC with both PDA and FLR detection alongside a photochemical reactor for enhanced detection and post-column derivatisation, which would therefore not be ideal for routine analysis [186]. Other examples include the determination of HT-2 and T-2 in cereals using LC-FLR, the study of T-2 and HT-2 toxins in cereals and cereal-based products including barley, the analysis of HT-2 and T-2 toxins in oats using LC-PDA and the determination of T-2 and HT-2 in oats using IAC and LC-FLR [139,140,187,188]. All of the aforementioned analyses meet the requirement of the EU Commission, which states that the LOD of the analytical method should be less than or equal to 25 µg/kg for the sum of T-2 and HT-2 [53].

Although the above methods are suitable, there are issues with the complexity of the analysis due to derivatisation for GC and FLR analyses, with an additional issue being that UV/PDA is not ideal due to neither molecule containing strong chromophores. Because of this, the majority of modern techniques for mycotoxin confirmation and quantification in cereals and cereal-derived foodstuffs use LC-MS, with both LC-MS/MS and LC-HRMS performed [128]. The use of such instrumentation allows the analysis of the analytes of choice, but facilitates the simultaneous analysis of modified or “masked” forms such as T2- and HT-2 glucosides, which, although they are not covered by legislation, may be of interest due to potentially being converted back to their native forms whilst traversing the gastrointestinal tract [183]. Various detectors coupled to LC systems have been employed for the confirmatory analysis of T-2 and HT-2, such as triple-quadrupole instruments (QqQ or MS/MS), high-resolution instruments such as orbital ion traps (Orbitrap), time-of-flight (TOF) and hybrid systems such as quadrupole-ToF (QToF), and even ambient MS such as direct analysis in real time (DART) [75,161,162,189,190].

The use of LC-MS has become the gold standard for the confirmatory analysis of mycotoxins, with the number of analytes in methods increasing over time due to improvements in the electronics of QqQ instrumentation facilitating faster cycle and dwell times, permitting the inclusion of more analytes. One very recent example of this is the study conducted by Sulyok et al. [122], which analysed more than 500 secondary microbial metabolites including T-2 and HT-2 toxins [122]. Another reason for increased analyte number is use of dilute-and-shoot (DnS) for sample clean-up, which allows the incorporation of numerous analytes due to the technique not being selective or specific due to having no stationary-phase-based chemistry. The creation of multi-methods allows numerous analytes to be incorporated, which in the case of analysing T-2 and HT-2 is not necessary as it is only those two analytes which must be reported on. However, as mentioned previously, it may be important to analyse other forms of these toxins such as their glucosides that may add to their overall toxicity on ingestion, as well as other metabolites such as T-2 triol and T-2 tetraol. Analysing for other *Fusarium* mycotoxins such as DON and ZEN, as well as some of the more important emerging mycotoxins according to EFSA, may also be advantageous, as although they are not regulated at present, routine analysis may highlight emerging threats brought about by climate change, which may aid the implementation of guidelines or regulatory levels. Furthermore, as indicated in the study by Kolawole et al. [79], the type of farming, crop season and harvest date all are factors that can influence mycotoxin prevalence and concentration and this may alter the mycotoxin profile as environmental changes occur over time [79].

Over the years, there have been numerous studies using LC-MS for the confirmatory analysis of T-2 and HT-2, with the majority not specific for T-2/HT-2 only, but rather incorporating other “relevant” mycotoxins depending on the matrix analysed. Although the focus of this review is oats and barley as foodstuffs for human consumption, these commodities are also used in the formulation of complete feed for ruminants, pigs and horses [144]. There has also been an increase in the use of oats for various other food products, such as in the production of oat milk and oat yoghurt. In order to analyse oats, barley, feed or cereal-based food products based on these, some sort of sample clean-up may be applied after extraction to remove unwanted matrix components that may interfere with the performance of the analytical method. These include the use of techniques listed in the section above, such as SPE, IAC, QuEChERS or modified forms of this, and dilute-and-shoot (DnS), which all serve to lower the LOD and LOQ of the analytical method. Juan et al. [159] developed a method using C18 SPE and LC-MS/MS for the measurement of sixteen mycotoxins in plant-based milks including oats. The limits of quantification for T-2 and HT-2 in oat milk were determined to be 4.2 and 5.3 µg/kg, respectively, and the repeatability and reproducibility were within the accepted limits according to EC directive 2002/657 [147].

There are many examples in use of LC-MS in the analysis of T-2 and HT-2 for oats, barley, cereal-based foods and animal feed. These include the study by Gottschalk et al. [130] for the analysis of trichothecenes in oats and oat products including T-2 and HT-2, as well as the T-2 metabolites T-2 triol and T-2 tetraol using LC-MS/MS and MycoSep SPE columns. The analysis was performed on an API 4000 (Applied Biosystems) in electrospray positive ionisation (ESI+) mode, with a run time of 35 min. The authors did not stipulate the LODs of the individual analytes, but they were stated to be in the range of 0.01–0.3 µg/kg for T-2 and HT-2. Interestingly, their study highlighted that T-2 tetraol played a major role in the overall contamination due to the mean levels being higher than those of T-2, concluding that it should be routinely monitored [130]. Another method developed by Lattanzio et al. [190] was validated using LC-MS/MS alongside SPE for the analysis of cereal-based foods, including barley and oats. Their method used a QTrap MS (Applied Biosystems) operated in both ESI+ and ESI- modes, simultaneously analysing 9 mycotoxins, including those of interest. The analysis time was >30 min for the 9 analytes, although 13C-labelled internal standards of each mycotoxin were also incorporated, with LODs of 0.5 and 1.1 µg/kg for T-2 and HT-2, respectively, in barley flour, and LODs of 1.2 and 2.4 µg/kg for T-2 and HT-2, respectively, in oat flour [190]. Another study by Soleimany et al. [186] used DnS alongside LC-MS/MS for the analysis of several (11) mycotoxins including T-2 and HT-2 in commercial cereals, including barley and oats. Their analysis was carried out in both ESI+ and ESI- modes with a 25 min run time, achieving an LOD and LOQ for both toxins of 5 and 10 µg/kg, respectively [186]. The LOD of this methodology is higher than those previously discussed, most likely due to the use of DnS as the clean-up step instead of SPE or IAC; however, the overall analytical method still meets the performance criteria as established by EC for analytical methods [117]. Kovač et al. [160] also applied DnS with LC-MS/MS to the analysis of the eleven regulated mycotoxins in maize, wheat and barley. As with Soleimany et al. [186], both ESI+ and ESI- modes were used but the run time was faster, at 18 min. The LOD and LOQ values for both T-2 and HT-2 toxins were determined to be 3 µg/kg and 10 µg/kg, respectively [160].

In 2017, a study by Annunziata et al. [142] developed a “fast, easy and cheap” method for the analysis of 8 mycotoxins in cereal-derived products intended for human consumption, including barley. They used a QuEChERS-based clean-up alongside LC-MS/MS, with analysis performed on an API 3000 QqQ MS (Applied Biosystems) in ESI+ and a run time of 30 min. The method was validated with LODs and LOQs of 1.3 and 2.5 µg/kg for both toxins, respectively, with the method applicable for use in both official and research facilities [142]. More recently, a modified QuEChERS sample preparation followed by LC-MS/MS for the simultaneous analysis of twenty-three mycotoxins was reported by Kim et al. [127]. Analysis was performed on an QTRAP^®^4500 mass spectrometry system (SCIEX, Darmstadt, Germany) in ESI+ and ESI- modes and the run time was 30 min. LODs of 0.16 µg/kg and 0.09 µg/kg were found for HT-2 toxin in barley and oats, respectively, while for T-2 toxin, the LOD was determined as 0.24 µg/kg for both barley and oats. The LOQs for barley and oats were determined as 0.52 µg/kg and 0.31 µg/kg (HT-2) and 0.79 µg/kg and 0.81 µg/kg (T-2). With intra-day and inter-day precisions of <10%, the method was shown to be applicable for routine analysis [127]. Furthermore, a survey carried out between 2013 and 2019 of mycotoxins in oats for food consumption by Meyer et al. [56] aimed to improve the database on the occurrence of specific mycotoxins in milling oats due to ongoing discussions in the European Commission on regulatory limits for certain mycotoxins. Their study looked at the 16 predominantly occurring trichothecenes including T-2 and HT-2 toxins in 281 commercial milling oats samples across 11 European provenances. Their analysis was carried out using an Agilent 6495 MS/MS in both ESI+ and ESI- modes using a MycoSpin SPE cartridge for sample clean-up, with an overall run time of 11 min, making this method more rapid than any of the aforementioned, with an LOQ of 5 µg/kg for both T-2 and HT-2. The mean concentration of the sum of T-2 and HT-2 toxins was 149 µg/kg, while the highest contamination found was a sample from Ireland, with a level of 1290 µg/kg for the sum of T-2 and HT-2 [56].

In 2021, two studies on oats from the island of Ireland were performed, with analysis performed using LC-MS/MS and a QuEChERS-based approach [78,79]. The methodology used by De Colli et al. [78] analysed 42 mycotoxins including T-2, HT-2, T-2-triol and T-2-glucoside (T2G) and also included 13C-labelled internal standards of T-2 and HT-2. The analysis was carried out on a Waters Quattro Premier XE QqQ MS operated in both ESI+ and ESI- modes, with the majority of analytes separated using a 15 min gradient. One note on this method was that the supernatant was split after the centrifugation step due to the response for some non-polar analytes being very intense compared to other analytes, resulting in saturation of the MS signal and linearity issues. Therefore, these were analysed on a separate four-minute gradient without concentration of the extract, but did not include the analytes of interest. Of those mycotoxins identified, several of the major type-A trichothecenes were present including HT-2 and T-2 toxins, T-2 triol and T-2-glucoside, with the most frequently quantified being HT-2 (51%) and T-2 (41%) toxins, with gluten-free oats containing significantly lower concentrations of HT-2 compared to conventionally produced oats. As mentioned previously, this work highlights the need for the routine testing of oats with multi-analyte methods to generate knowledge on the occurrence of other mycotoxins that are to date rarely investigated. The study by Kolawole et al. [79] was less comprehensive in its suite of toxins, but included both T-2 and HT-2 along with other regulated type-B trichothecenes, DON and ZEN. This methodology was rapid, with separation in under 7 min using a SCIEX 5500+ QqQ (AB SCIEX). Initially, a simple DnS approach was applied after extraction with acetonitrile:water:acetic acid (79:20:1, *v*/*v*/*v*); however, the extract was not suitable for injection even after filtration. Instead, a QuEChERS-based approach was used but without the addition of any dSPE. Furthermore, this method has been extended in-house to include other regulated mycotoxins such as the aflatoxins B, B1, G and G1, ochratoxin A (OTA) and fumonisins B1 (FB1) using the same analytical conditions.

There are other methodologies that use LC-HRMS, and although the newer hybrid-systems have the quadrupole functionality for quantification, they are not routinely used for confirmatory quantitative analysis. These systems are also not ideally suited to the analysis of only a few analytes, such as in the case of the methodology required. Instead, they are more routinely used in the qualitative analysis of sample extracts for numerous analytes across different classes, with ESI+ and ESI- modes used. Due to the differing polarities of mycotoxins and indeed other potential contaminants from different classes, the gradients and therefore run times used in LC-HRMS are usually longer in order to achieve better resolution of adjacent peaks. This, along with running the extracts in both polarities, serves to increase the run-time of the methodology. Once the data are generated, databases that are generated either in-house or provided online by the various vendors are used to search for various contaminants, including mycotoxins. This type of approach is usually considered non-targeted; however, with the matrix and an idea of the potential contaminants known, this approach can be considered targeted untargeted. An advantage of HRMS over QqQ systems is the ability to retrospectively mine the data generated from a sample extract for emerging threats to see if it was present. A further advantage for the use of LC-HRMS is that the confirmation of analytes can be performed through the use of databases.

Some examples of the use of LC-HRMS for T-2 and HT-2 include the studies by Tamura et al. [123] and Romera et al. [157]. The former analysed 20 *Fusarium* toxins in cereals including barley, using an LC-Orbitrap MS with a two-step clean-up, which included the use of QuEChERs followed by purification using a Multistep 229 Ochra multifunctional cartridge. This was performed in order to lower the detection level of the method, as HRMS instruments are generally not as sensitive as QqQ instruments. However, for routine analysis, this is not cost-effective. The latter study used UPLC-MS/MS for the simultaneous analysis of numerous mycotoxins in compound feed for swine, sheep, poultry, cattle and equines, with these confirmed by the UPLC-QToF, with many other fungal metabolites (mycotoxins) also identified through analysis using UPLC-QToF. This approach indicates that there may be many more mycotoxins present than are covered by the LC-MS/MS method, which in itself is targeted to the analytes of interest. In this case, screening a sample extract using LC-HRMS may be beneficial, as it can provide direction for the analyst as to which quantitative method should be applied; however, again, this is not cost-effective, especially in relation to the analysis of the target analytes covered by this review. From a research standpoint though, both methods are useful for the analysis of the target commodities for the analytes of choice.

## 8. Discussion

This review aimed to evaluate the current information relating to the toxicity, occurrence, biosynthesis, impact of climate change, and analytical techniques available for the accurate determination of T-2 and HT-2 toxins in cereals, particularly oats and barley.

In terms of the toxicological effects of these toxins in animals, poultry and pigs are particularly sensitive to T-2 toxin. That said, in all species assessed, consumption of this toxin resulted in feed refusal, irritation/lesions/haemorrhaging of the intestinal mucosa, dermatitis and oral lesions. T-2 toxin has also been shown to be an endocrine disruptor affecting egg production and shell quality in poultry and to have induced infertility and abortion in pigs and ruminants. The immunosuppressive effects of this toxin have been detailed in many animal species, including leucopoenia and increased likelihood of infection in poultry, a reduction in white blood cells in piglets and sheep, alteration of immunoglobulin serum concentrations and a reduction in neutrophil function and lymphocyte blastogenesis in ruminants. In addition, alterations of serum enzyme concentrations have been observed in rabbits and in cats, and lymphatic tissues changes were noted.

Few reports of human poisoning have been reported, except for alimentary toxic aleukia (ATA), which caused the death of 60% of those who consumed T-2 toxin and DAS-contaminated grain. Similar clinical symptoms in animals have included gastroenteritis, abdominal and oesophageal pain, leucopoenia, lymphocytosis, and severe bleeding of the nasal, oral, gastric and intestinal mucosa. T-2 toxin has also been linked to Kashin–Beck disease, a joint disease; however, there is a lack of epidemiological studies to support the link to T-2 toxin. Finally, although T-2 toxin was implicated in the so-called “yellow rain” attacks, no definitive proof was presented that it was used in biological warfare.

Many pathogenic *Fusarium* species produce T-2 and HT-2 toxins, such as *F. graminearum*, *F. culmorum*, *F. poae*, *F. avenaceum*, *F. sporotrichioides*, *F. langsethiae* and *F. nivale*. The prevalence of toxigenic *Fusarium* species producing T-2 and HT-2 toxins is largely dependent on the geographical region and climatic conditions from one year to the next, with temperature and moisture being the most important factors affecting the colonisation of cereals and contamination with mycotoxins. In European countries, oats are frequently contaminated with high concentrations of T-2 and HT-2 toxins. Furthermore, agronomic practices heavily influence the contamination levels of T-2 and HT-2 toxin in oats. The biosynthetic pathways for the production of T-2 and HT-2 toxins are well elucidated and share similar enzyme reactions; however, depending on the strain, chemotype and geographical distribution, other trichothecene mycotoxins such as DON and NIV may be produced as a result of allelic variations in the TR11 gene.

The extraction techniques employed for the analysis of T-2 and HT-2 toxins include liquid–liquid, solid–liquid, ultrasonic or accelerated solvent extraction depending on the matrix and analysis. Typically, a mixture of organic solvent with water has been used (i.e., acetonitrile or methanol and water); however, aqueous buffers have also been applied for the more rapid test kits. Solid-phase extraction, immunoaffinity columns and QuEChERS have all successfully been employed for sample clean-up. With many of the confirmatory methods now applied having the ability to simultaneously measure multiple mycotoxins, care must be taken with the selection of the SPE. This issue also arises with the use of IACs; several may be needed, depending on the mycotoxins being analysed, which would not be very cost effective. The use of QuEChERS has grown dramatically for mycotoxin analysis and has the advantage over SPE and IAC of being more cost-effective. Another technique commonly used is dilute-and-shoot, whereby the sample extract is diluted and filtered prior to analysis. This method has been applied in multi-methods and while it is advantageous in terms of cost, the matrix is simply reduced and LOQs will be compromised as a result.

A broad range of analytical methods exist for the determination of T-2 and HT-2 toxins in cereals, food and feed including both screening and confirmatory tests. The majority of screening assays available for their analysis are based on immunological methods such as LFDs, ELISA, FPIA, biosensors and SERS. The advantages of these tests include their easy handling, that they are rapid/convenient, require few procedural steps, are portable, provide the required sensitivity, and may be quantitative or qualitative; in addition, many have water-based extractions, limiting the use of solvents. The disadvantages associated with these rapid tests include antibody cross-reactivity. As the current indicative limits refer to the sum of T-2 and HT-2 toxins, the antibody must have good cross-reactivity with both toxins, otherwise under-estimation or over-estimation of results will be reported. Other limitations to these tests include interference by the matrix being tested, resulting in either under-estimation or over-estimation of results; that many still employ solvents for extraction; temperature dependency; and the fact that dust and dirt can affect readers. Discussions are ongoing within Europe as to the introduction of maximum regulatory limits for the sum of T-2 and HT-2, and the consensus is that the limits will be reduced. If this is the case, some of the rapid tests may require changes to ensure “fitness for purpose”.

In terms of confirmatory methods of analysis for T-2 and HT-2 toxins, significant advances have been seen over the years. Earlier techniques employed GC or LC (HPLC/UHPLC) coupled to FID, MS or PDA using UV or FLR; however, using GC-MS or HPLC-FLR necessitated derivatisation steps, thus adding complexity and potential errors into the methodology. For HPLC-UV/PDA, due to the lack of a strong chromophore, these methods are only applicable to those samples containing high contamination levels. Therefore, in view of this, modern methods of analysis typically use LC-MS or LC-HRMS. Targeted LC-MS provides the sensitivity required and allows the simultaneous determination of many mycotoxins and is therefore seeing increased use for routine analysis. LC-HRMS, on the other hand, although not suitable for routine analysis due to the high costs, is beneficial in revealing what metabolites may be present in the commodities tested, thus providing information on which analytes should be targeted for routine testing.

## 9. Conclusions

T-2 toxin and its metabolite HT-2 toxin are detrimental to the health of animals and humans alike. These toxins are potent inhibitors of protein synthesis. Moreover, they are immunosuppressive and dermatotoxic, causing necrosis and haemorrhage of the intestinal mucosa. The clinical symptoms of T-2/HT-2 mycotoxicoses in animals include weight loss, decreased feed conversion and feed refusal, vomiting, diarrhoea, skin problems, haemorrhage, decreased egg production, abortion and death. With respect to human health, these mycotoxins have been associated with several poisonings, the most significant being alimentary toxic aleukia that caused the death of 60% of those infected in the former U.S.S.R. from 1932 until 1947. Furthermore, although the aetiology has not been confirmed, it is believed that T-2 may play a causal role in Kashin–Beck disease (KBD). In addition, it has been implied that T-2 toxin has been used in biological warfare, although these claims have been disputed in the scientific literature.

In terms of the indicative limits applied to the sum of T-2 and HT-2, discussions are ongoing as to the implementation of maximum limits that will be much lower than those currently adhered to. For example, for cereals for direct human consumption, the limits could change from 200 µg/kg to 50 µg/kg for oats and from 50 µg/kg to 20 µg/kg for other cereals. This would have a serious economic impact on the cereal industry, not least on the Island of Ireland, where the main crops produced are oats and barley. Increased surveillance and mitigation strategies would add an extra burden on farmers and producers.

Further research is required on the ecology of the *Fusarium* species producing T-2 and HT-2 toxins. Furthermore, the environmental factors that influence the growth and activation of the biosynthetic genes responsible for these toxins are still not fully elucidated. This information will be vital to help predict contamination and allow early interventions to reduce contamination.

Many analytical methods exist for the determination of T-2 and HT-2 toxins in foods and feeds. Screening assays are hugely important for on-site testing to give rapid results of compliance/non-compliance. As such, these tests must be easy to perform and interpret. Many rapid diagnostic kits are available commercially, and farmers/producers should take advantage of these to monitor their crops. Of course, confirmatory analysis must be performed in a laboratory setting using sophisticated technology. The state-of-the-art is LC-MS that allows the simultaneous determination of multiple mycotoxins, thereby identifying the mycotoxins and concentrations that may pose a risk to humans and animals. Moreover, distribution patterns of mycotoxins due to changing climatic conditions and agronomic factors could be identified, thereby providing useful information for mitigation. The use of high-resolution mass spectrometry to characterize metabolites of T-2 and HT-2 and emerging mycotoxins would be advantageous in predicting what testing would be required under a changing climate and thus promote a proactive approach to the continued risk of mycotoxin contamination in cereal crops.

## Figures and Tables

**Table 1:** Specific side chains of trichothecene mycotoxins of interest.

Trichothecene	R1	R2	R3	R4	R5
T-2 Toxin	-OH	-OCOCH_3_	-OCOCH_3_	-H	-OCOCH_2_CH(CH_3_)_2_
HT-2 toxin	-OH	-OH	-OCOCH_3_	-H	-OCOCH_2_CH(CH_3_)_2_

**Table 2:** Lowest observed adverse effect levels (LOAELs) in a range of animal species.

Animal	LOAEL (µg T-2 Toxin/kg b.w. Per Day)
Pigs	29
Calves, lambs	300
Ruminants	Not identified
Poultry	40
Laying hens	120
Broiler chickens	48
Fattening ducks	40
Fattening turkeys	48
Rabbits	200–500
Catfish	13
Cats	Not identified
Horses	Not identified

Data from the European Food Safety Authority (EFSA) [15].

**Table 3:** EU indicative levels and maximum limits under discussion for cereals and cereal products.

Commodity	Indicative Levels for the Sum of T-2 and HT-2 (μg/kg)	Maximum Limits under Discussion
Unprocessed cereals:		
Barley (including malting barley) and maize	200	100
Oats (with husk)	1000	500
Wheat, rye, other cereals	100	50
Cereals for direct human consumption:		
Oats	200	50
Maize	100	50
Other cereals	50	20
Cereal products for human consumption:		
Oat bran and flaked oats	200	50
Cereal bran except oat bran, oat milling products other than oat bran and flaked oats, and maize milling products	100	50
Other cereal milling products	50	20
Breakfast cereals including formed cereal flakes	75	20
Bread (including small bakery wares), pastries, biscuits, cereal snacks, pasta	25	10
Cereal-based foods for infants and young children	15	10
Cereal products for feed and compound feed:		
Oat milling products (husks)	2000	
Other cereal products	500	
Compound feed, except feed for cats	250	

**Table 5:** Instrument platforms and extraction techniques employed for the identification and determination of T-2 and HT-2 toxins, using both screening and confirmatory analyses.

Detection Platform Used	Matrices Analysed	Extraction and Clean-Up Method	Analyte(s) Included	LOD/LOQ [T-2/HT-2 Only]	Type of Analysis	Reference
LC-MS/MS: Shimadzu LC–MS 8050 triple-quadrupole MS equipped with a Nexera X2 UHPLC (Shimadzu, Kyoto City, Japan)	Feed ingredients and compound feed	QuEChERS: Extraction with 10 mL ACN, then 10 mL Water (10% formic) followed by dSPE: C_18_ and PSA	11 mycotoxins including T-2 and HT-2 toxins	Not detailed	Confirmatory/quantitative	[121]
LC-HRMS: Ultimate 3000 LC coupled to a Q-Exactive™ Orbitrap MS (Thermo Fisher Scientific, Waltham, MA, USA)	Cereals (corn, wheat and barley)	QuEChERS + SPE: Extraction with Water:ACN (50:50, *v*/*v*) containing 2% formic acid, followed by QuEChERS + Multistep 229 Ochra Cartridge	20 fusarium toxins including T-2 and HT-2 toxins	LOQ: 5 µg/kg for HT-2 and T-2 toxins	Confirmatory/quantitative	[123]
^1^ELISA: Ridascreen T-2/HT-2 R3805 (R-Biopharma) with a Multiskan™ FC microplate photometer reader (85 and 100% cross-reactivity with antibody used, respectively) ^2^UPLC-FLD: Acquity UPLC H-Class Bio System coupled to a FLR detector (Waters, Milford, MA, USA)	Cereals (barley, malting barley, maize, oats, wheat and rye)	^1^ELISA: Extraction buffer only ^2^IAC: Extraction with 90% MeOH_(aq)_	T-2 and HT-2 toxins	^1^LOD: 75 µg/kg (sum of T-2/HT-2) ^2^LOQ: 29 and 19 µg/kg (T-2 and HT-2 respectively)	^1^Screening/qualitative ^2^Confirmatory /quantitative	[125]
GC-MS: Agilent GC 6890 equipped with an inert 5973 N mass selective detector with EI ionisation	Processed cereal-based baby foods	QuEChERS: Extraction with 15 mL water, then 10 mL can followed by either: 1. dSPE: C_18_ and PSA 2. IAC 3. MultiSep 226 clean-up column (SPE)	12 mycotoxins including T-2 and HT-2 toxins	LOD: 6.4 and 6.76 µg/kg (HT-2 and T-2 respectively) LOQ: 21.1 and 22.3 µg/kg (HT-2 and T-2 respectively) NB: Method 1 only	Screening/qualitative	[150]
UPLC-PDA: Acquity UPLC^®^ system equipped with a PDA detector (Waters)	Oats and wheat	IAC: Extraction with 90% MeOH_(aq)_	T-2 and HT-2 toxins only	LOD: 8 µg/kg for both T-2 and HT-2 in both matrices	Confirmatory/quantitative	[139]
ELISA: Competitive AlphaLISA using a SpectraMax I3 Microplate reader (Molecular Devices) (No cross-reactivity with the antibody used specified)	Flour, cornmeal and formulated feed.	LLE: Extraction with 70% MeOH_(aq)_ then dilution with 70% MeOH_(aq)_ (No further clean-up)	T-2 and HT-2 toxins only	LOD: 0.03 ng/mL for T-2 and HT-2 toxins	Screening/qualitative	[151]
APCI-MS: TD-APCI-MS (Portable MS)	White wheat, red wheat, and yellow dent maize	SPE: Extraction with ACN:Water (84:16, *v*/*v*) then MycoSep 225 clean-up column	T-2 and HT-2 toxins only	LOD: 28 and 20 µg/kg for T-2 in wheat. LOD: 2.0, 1.5 and 0.9 mg/kg for HT-2 in wheat and maize respectively.	Screening/qualitative	[152]
FPIA: HT-2 specific antibody using a Sentry^®^ 100 portable reader (Diachemix Corporation) (80% cross-reactivity for T-2 and both T-2/HT-2 glucosides)	Wheat	LLE: Extraction with MeOH:Water (9:1, *v*/*v*) or water only followed by filtration	T-2, HT-2 and their glucosides	LOD and LOQ: 10 and 15 µg/kg respectively for all analytes	Screening/qualitative	[153]
iSPR: Mult17mmuneimmuno-assay with nanostructured iSPR chips. Performed on a Biacore 3000 SPR instrument (GE Healthcare) (76% cross-reactivity for HT-2)	Barley	LLE: Extraction with 80% MeOH_(aq)_ then dilution with HBS-EP buffer to 20% MeOH_(aq)_	T-2 and HT-2 toxins only (76% cross-reactivity for HT-2)	LOD: 26 µg/kg (T-2 only)	Screening/qualitative	[154]
GC-MS/MS: Agilent 7890A coupled. with an Agilent 7000A QqQ MS with inter electron-impact ion source LC-MS/MS: Agilent 1200 LC system coupled to a 3200 QTRAP^®^ QqQ MS (ABSCIEX, Framingham, MA, USA)	Various commodities including: ^a^cereals, ^b^legumes, ^c^fish, ^d^vegetables and ^e^meats.	QuEChERS: Extraction with 10 mL water (2% formic) then 10 mL ACN, followed by QuEChERS (dSPE: C_18_) NB: Derivatisation of sample before GC-MS analysis	26 mycotoxins including T-2 and HT-2 toxins	^a, b, c, e^LOD: 0.75 and 0.15 µg/kg (T-2 and HT-2 respectively) ^d^LOD: 0.75 µg/kg for T-2 and HT-2 toxins	Confirmatory/quantitative	[155]
LC-MS/MS: 1100 series micro-LC (Agilent) coupled to a QTrap QqQ MS (ABSCIEX)	Cereals and cereal-based food	IAC: Extraction with water followed by MeOH. Samples filtered then diluted with PBS.	26 mycotoxins including T-2 and HT-2 toxins	LOQ: 5 µg/kg for T-2 and HT-2 toxins	Confirmatory/quantitative	[137]
LC-HRMS: Exactive™ Orbitrap MS equipped with a heated ESI source (HESI II) coupled to an Accela HPLC system (Thermo Fisher, Waltham, MA, USA)	Barley including malt	SPE: Extraction with ACN:Water (84:16, *v*/*v*) then MycoSep 225 clean-up column	T-2, HT-2 and their glucosides (T2G and HT2G)	LOQ: 5 µg/kg for T-2 and HT-2 toxins; 0.1 µg/kg for T2G and HT2G	Confirmatory/quantitative	[137]
LC-MS/MS: API 3000 QqQ MS (Applied Biosystems) coupled to a model series 200 HPLC system (Perkin Elmer, Waltham, MA, USA)	Cereal-derived products	QuEChERS: Extraction with 10 mL of water (0.1% formic) then 10 mL ACN. (No dSPE used)	8 mycotoxins including T-2 and HT-2 toxins	LOD: 1.3 µg/kg for T-2 and HT-2 toxins LOQ: 2.5 µg/kg for T-2 and HT-2 toxins	Confirmatory/quantitative	[142]
LC-MS/MS: Acquity UHPLC system coupled to a Waters Quattro Premier XE QqQ MS with ESI probe (Waters)	Oats	QuEChERS-based protocol: Extraction with 10 mL of a 1% aqueous acetic acid solution then 10 mL ACN. (No dSPE used)	42 mycotoxins including T-2, HT-2, T2G and T2-3OH	LODs: 12.1 and 17.2 µg/kg (T-2 and HT-2 respectively) 4.1 and 6.5 µg/kg (T2-3OH and T2G respectively)	Confirmatory/quantitative	[78,156]
LC-MS/MS: A 1290 Infinity UHPLC system coupled to a 6460 QqQ MS (Agilent, Santa Clara, CA, USA)	Seven raw materials and eight animal feeds	QuEChERS-based protocol: Extraction with 10 mL of a 2% aqueous acetic acid solution then 10 mL ACN. (No dSPE used)	22 mycotoxins including T-2 and HT-2	LOQ: 2.7 and 14.3 µg/kg (T-2 and HT-2 toxins respectively)	Confirmatory/quantitative	[144]
LC-MS/MS: A 1290 Infinity UHPLC system coupled to a 6495 QqQ MS (Agilent)	Oats	SPE: Extraction with 20 mL ACN:Water:Acetic Acid (79:20:1, *v*/*v*/*v*), then SPE using a MycoSpin^TM^ 400 SPE cartridge.	16 mycotoxins including T-2 and HT-2	LOQ: 5 µg/kg for T-2 and HT-2 toxins	Confirmatory/quantitative	[56]
^1^LC-MS/MS: ACQUITY UPLC™ system coupled to an ACQUITY TQD tandem quadrupole MS ^2^LC-HRMS: ACQUITY UPLC™ system coupled to a Triple TOF 5600 System (AB SCIEX, Framingham, MA, USA)	Feed samples: swine, sheep, poultry, cattle, equine, aquaculture and feed materials	DnS: Extraction with 8 mL ACN:Water:Formic Acid (80:20:1, *v*/*v*/*v*) then filtered	15 mycotoxins including T-2 and HT-2	LOD: 12.5 µg/kg for T-2 and HT-2 toxins (matrix not specified)	^1^Confirmatory/quantitative ^2^Screening	[157]
LC-MS/MS: QTRAP 6500^+^ ultra-HPLC-MS/MS instrument equipped with an ESI source (AB SCIEX)	Maize	Multiple-impurity adsorption purification (MIcan) Extraction with 70% ACN_(aq)_ then MIA added to an aliquot for purification	11 mycotoxins including T-2 and HT-2	LOD: 0.2 and 0.8 µg/kg for T-2 and HT-2 toxins	Confirmatory/quantitative	[158]
LC-MS/MS: HPLC Nanospace SI-2 (Shieido, Tokyo, Japan) coupled to a QTRAP^®^4500 mass spectrometry system (SCIEX, Darmstadt, Germany)	Wheat, oat and barley	QuEChERS-based protocol: Extraction with 10 mL of distilled water then 10 mL of 5% formic acid in ACN (No dSPE used)	23 mycotoxins including T-2 and HT-2	LOD: T-2: 0.24 µg/kg for oats and barley HT-2: 0.16 µg/kg for barley and 0.09 µg/kg for oats. LOQ: T-2: 0.79 V and 0.81 V for barley and oats HT-2: 0.52 µg/kg and 0.31 µg/kg in barley and oats	Confirmatory/quantitative	[127]
LC-MS/MS: Agilent 1200 LC binary pump chromatograph and autosampler, coupled to 3200 QTRAP^®^ AB SCIEX (Applied Biosystems, Foster City, CA, USA)	Oat, rice, soy and almond drinks	SPE: Extraction with 5 mL ACN then SPE using a STRATA^®^ C18-E column.	16 mycotoxins including T-2 and HT-2	LOD (oat drink): T-2: 0.8 µg/kg HT-2: 1.1 µg/kg LOQ (oat drink): T-2: 4.2 µg/kg HT-2: 5.3 µg/kg	Confirmatory/quantitative	[159]
LC-MS/MS: UHPLC (Acquity H-Class, Waters, Milford, MA, USA) coupled to a triple quadruple mass spectrometer (XEVO TQD, Milford, MA, USA)	Maize, wheat and barley	DnS: Extraction with acetonitrile/water/formic acid (79:20:1, *v*/*v*/*v*) then a dilution using water/acetonitrile/formic acid (79:20:1, *v*/*v*/*v*)	11 mycotoxins including T-2 and HT-2	LOD (barley): T-2 and HT-2: 3 µg/kg LOQ (barley): T-2 and HT-2: 10 µg/kg	Confirmatory/quantitative	[160]
LC-MS/MS: Sciex QTRAP 4500 tandem quadrupole mass spectrometer coupled to an ExionLCTM AC LC system	Animal feed	IAC: Extraction with 20mL ACN–H_2_O– HCOOH (79:20:1, *v*/*v*/*v*) followed by IAC clean-up using 11+Myco MS-PREP^®^	11 mycotoxins including T-2 and HT-2	LOD: T-2 and HT-2: 0.2 µg/kg and 1 µg/kg LOQ: T-2 and HT-2: 0.7 µg/kg and 3 µg/kg	Confirmatory/quantitative	[138]
LC-HRMS: Dionex UltiMate 3000 UHPLC coupled to a Thermo Scientific Q-Exactive Plus Orbitrap mass spectrometer (Thermo Scientific, San Jose, CA, USA)	Corn and wheat	QuEChERS-based protocol: Extraction with 4 mL of MeCN:H_2_O:acetic acid (79:20:1, *v*/*v*/*v*) (No dSPE used)	11 mycotoxins including T-2 and HT-2	LOD (corn): T-2: 4.8 µg/kg HT-2: 43 µg/kg LOQ (corn): T-2: 10 µg/kg HT-2: 64 µg/kg LOD (wheat): T-2: 11 µg/kg HT-2: 14.5 µg/kg LOQ (wheat): T-2: 15 µg/kg HT-2: 20 µg/kg	Confirmatory/quantitative	[161]
LC-MS/MS: HPLC (Agilent Technologies) coupled to an Agilent G6410A triple quadrupole (QqQ) mass spectrometer	Grass	Dispersive magnetic solid-phase extraction (DMSPE): Extraction with 10 mL distilled water containing 2% *m*/*v* NaCl and 400 μL Fe_3_O_4_@ PPy microcomposite suspension	13 mycotoxins including T-2 and HT-2	LOD: T-2: 5.3 µg/kg HT-2: 11 µg/kg LOQ: T-2: 17 µg/kg HT-2: 37 µg/kg	Confirmatory/quantitative	[135]
LC-HRMS: Dionex UltiMate^®^ 3000 system UPLC coupled to Q-Exactive Orbitrap	Breakfast cereals	Extraction with 10 mL of ACN containing 0.1% formic acid followed by salting out with NaCl	24 mycotoxins including T-2 and HT-2	LOQ: T-2: 0.39 µg/kg HT-2: 0.78 µg/kg	Confirmatory/quantitative	[162]

ACN: acetonitrile; APCI: atmospheric pressure chemical ionisation; LC: liquid chromatography; ESI: electrospray ionisation; UPLC: ultra-performance liquid chromatography; UHPLC: ultra-high-performance liquid chromatography; MeOH: methanol; QqQ MS: triple-quadrupole mass spectrometry, QuEChERS: quick, easy, cheap, effective, rugged and safe, SPE: solid-phase extraction; dSPE: dispersive solid-phase extraction; HPLC: high-performance liquid chromatography; LOD: limit of detection; LOQ: limit of quantification; iSPR: imaging surface plasmon resonance; LC-MS/MS: liquid chromatography tandem mass spectrometry; MIA: multiple-impurity adsorption purification; IAC: immunoaffinity chromatography; ELISA: enzyme-linked immunosorbent assay; LC-HRMS: liquid chromatography high-resolution mass spectrometry; FLD/FLR: fluorescence detection; PDA: photodiode array; GC-MS: gas chromatography mass spectrometry; GC-MS/MS: gas chromatography tandem mass spectrometry; LLE: liquid–liquid extraction; EI ionisation: electron impact ionisation; FPIA: fluorescence polarisation immunoassay. ^1^ELISA, ^2^UPLC-FLD, ^a^cereals, ^b^leg-umes, ^c^fish, ^d^vegetables, ^e^meats.

**Table 6:** Commercially available rapid diagnostic tools for the detection of T-2/HT-2 in cereals.

Manufacturer	Kit	Matrix	Analytical Method	Detection Method	Extraction Solvent	Limit of Detection (LOD) (µg/kg)	Antibody Cross-Reactivity Profile	Test Time (Incubation Following Sample Preparation)
Aokin AG (Berlin, Germany)	Aokin Mycontrol T2/HT2	Oats, wheat, corn, other grains	FPIA	Quantitative	Methanol based SPE clean-up	Not detailed	Not detailed	15 min
Charm Sciences Inc. (Lawrence, MA, USA)	ROSA T-2 and HT-2 Quantitative Test	Barley, corn, corn gluten meal, oat groats, sorghum soybean meal, wheat, wheat flour	LFD	Quantitative	70% methanol	Not detailed	Not detailed	10 min
Elabscience Inc. (Houston, TX, USA)	T-2(T-2 Toxin) Lateral Flow Assay Kit	Cereals, feed	LFD	Qualitative	Ethyl acetate	10 µg/kg	Not detailed	5 min
Elabscience Inc. (Houston, TX, USA)	T-2(T-2 Toxin) ELISA Kit	Beans, corn, oats, peanuts, feed	ELISA	Quantitative	60% methanol	0.05 µg/kg	Not detailed	45 min
Elabscience Inc. (Houston, TX, USA)	T-2(T-2 Toxin) ELISA Kit	Cereals, feed	ELISA	Quantitative	70% methanol	1 µg/kg	T2: 100% ZEN: 59% HT2: <1%	20 min
Envirologix Inc. (Portland, ME, USA)	QuickTox Kit for QuickScan T-2/HT-2 Flex	Corn	LFD	Quantitative	Extraction buffer	25–50 µg/kg	AFB1: <1% DON: <1% FB1: <1% OTA: <1% ZEN: <1%	5 min
Eurofins Tecna Laboratories (Luxembourg City, Luxembourg)	B ZERO T2	Cereals, feed	ELISA	Quantitative	70% methanol and 4% NaCl	25 µg/kg Oats: 40 µg/kg	T2: 100% HT2: 72%	20 min
Eurofins Tecna Laboratories (Luxembourg City, Luxembourg)	Celer T2	Cereals, feed	ELISA	Quantitative	70% methanol and 4% NaCl	25 µg/kg Oats: 40 µg/kg	T2: 100% HT2: 72% DON: <1%	20 min
Hygiena LLC (Camarillo, CA, USA)	Helica™ T-2 Toxin ELISA	Cereals, feed	ELISA	Quantitative	70% methanol	12.5 µg/kg	T2: 100% HT2: 3%	30 min
Neogen Corporation (Lansing, MI, USA)	Reveal^®^ Q+ MAX for T-2/HT-2	Wheat, oats, corn	LFD	Quantitative	Aqueous extraction	50 µg/kg	Not detailed	5 min
Neogen Corporation (Lansing, MI, USA)	Reveal^®^ Q+ for T-2/HT-2	Corn, corn products	LFD	Quantitative	Water	50 µg/kg	Not detailed	6 min
Neogen Corporation (Lansing, MI, USA)	Veratox^®^ for T-2/HT-2	Barley, corn, corn flour, corn gluten, corn steep, DDGS wet cake, oats, oat hulls (whole), rice (brown), rice flour (white), rice gluten, rice hulls, rye, pea fibre, potato (white), soy, soybean meal, tapioca, wheat, wheat bran, wheat flour, wheat gluten	ELISA	Quantitative	70% methanol	25 µg/kg	T2: 100% HT2: 100%	10 min
R-Biopharm AG (Darmstadt, Germany)	RIDA^®^QUICK T-2/HT-2 RQS ECO	Oats, wheat, corn	LFD	Quantitative	Aqueous extraction buffer	50 µg/kg	Not detailed	5 min
R-Biopharm AG (Darmstadt, Germany)	RIDASCREEN^®^ T-2/HT-2 Toxin	Oats, corn, barley, wheat	ELISA	Quantitative	Water-based extraction	12 µg/kg	T2: 100% HT2: 85% T2 Triol: <0.5% T2 Tetraol: <0.5%	45 min
R-Biopharm AG (Darmstadt, Germany)	RIDASCREEN^®^ T-2 Toxin	Cereals, feed	ELISA	Quantitative	84% acetonitrile	3.5–56 µg/kg	T2: 100% HT2: 7% Acetyl T2: <114% Iso T2: 2%	90 min
R-Biopharm AG (Darmstadt, Germany)	RIDASCREEN^®^FAST T-2 Toxin	Cereals, feed	ELISA	Quantitative	70% methanol	<20 µg/kg	Not detailed	15 min
Romer Labs Diagnostic GmbH (Tulln, Austria)	AgraQuant^®^ T-2 Toxin ELISA test	Grains, cereals, other commodities	ELISA	Quantitative	70% methanol	10 µg/kg	Not detailed	15 min
Perkin Elmer Inc. (Waltham, MA, USA)	AuroFlow™ AQ T-2/HT-2 Strip Test	Corn, wheat	LFD	Quantitative	Water-based	50 µg/kg	Not detailed	5 min
	MaxSignal^®^ T-2 ELISA Kit	Milk, dried meat, dried fish, seed, feed, cereal	ELISA	Quantitative	Not detailed	10 µg/kg	Not detailed	<30 min
Vicam LP (Milford, MA, USA)	T2/HT2-V AQUA	Grains, feed	LFD	Quantitative	Water-based extraction	10 µg/kg	Not detailed	5 min

ELISA: enzyme-linked immunosorbent assay; LFD: lateral flow device/dipstick assay; FPIA: fluorescence polarisation immunoassay; µg/kg: parts per billion; SPE: solid-phase extraction.

## Data Availability

The data presented in this study are available in this article.
